# Glucocorticoid Receptor-mediated transactivation is hampered by Striatin-3, a novel interaction partner of the receptor

**DOI:** 10.1038/s41598-017-09246-6

**Published:** 2017-08-21

**Authors:** Ioanna Petta, Nadia Bougarne, Jolien Vandewalle, Lien Dejager, Sofie Vandevyver, Marlies Ballegeer, Sofie Desmet, Jonathan Thommis, Lode De Cauwer, Sam Lievens, Claude Libert, Jan Tavernier, Karolien De Bosscher

**Affiliations:** 1 Receptor Research Laboratories, Cytokine Receptor Lab, VIB, Center for Medical Biotechnology, Ghent, Belgium; 2 Department of Biochemistry, Ghent University, Ghent, Belgium; 3 Center for Inflammation Research, VIB, Ghent, Belgium; 4 Department of Biomedical Molecular Biology, Ghent University, Ghent, Belgium; 5 Receptor Research Laboratories, Nuclear Receptor Lab, VIB, Medical Biotechnology Center, Ghent, Belgium; 6 Cancer Research Institute Ghent (CRIG), Ghent, Belgium

## Abstract

The transcriptional activity of the glucocorticoid receptor (GR) is co-determined by its ability to recruit a vast and varying number of cofactors. We here identify Striatin-3 (STRN3) as a novel interaction partner of GR that interferes with GR’s ligand-dependent transactivation capacity. Remarkably, STRN3 selectively affects only GR-dependent transactivation and leaves GR-dependent transrepression mechanisms unhampered. We found that STRN3 down-regulates GR transactivation by an additional recruitment of the catalytic subunit of protein phosphatase 2A (PPP2CA) to GR. We hypothesize the existence of a functional trimeric complex in the nucleus, able to dephosphorylate GR at serine 211, a known marker for GR transactivation in a target gene-dependent manner. The presence of STRN3 appears an absolute prerequisite for PPP2CA to engage in a complex with GR. Herein, the C-terminal domain of GR is essential, reflecting ligand-dependency, yet other receptor parts are also needed to create additional contacts with STRN3.

## Introduction

Glucocorticoids (GC) exert effects at multiple levels of cellular functionality, including energy metabolism, cell fate and immune response. The majority of actions by hormonal GC ligands are mediated by the glucocorticoid receptor (GR), a transcription factor that can regulate gene expression in a positive or negative manner^1, 2^. To exert its gene regulatory effects, GR interacts, mainly via its ligand binding domain (LBD)^3^ with different proteins including other transcription factors, modifying enzymes, chromatin modulators, scaffold proteins, co-activators, co-repressors and co-chaperone proteins^4^.

GR-mediated gene promoter activation involves DNA binding of a homodimeric GR on a palindromic GC response element (GRE) in the promoter (simple GRE), from a coordinated DNA binding of a GR/transcription factor complex onto a so-called composite GRE or from a GR/transcription factor tethering mechanism. The latter two mechanisms can also form the basis for GR-mediated promoter repression^5, 6^. Recently, also a role for monomeric GR in direct target gene activation was discovered. More specifically, monomeric GR can bind to half sites and promote gene activation^7^. In an independent study using endogenous GCs, monomeric GR-binding to half-sites was found to be even more prevalent than homodimer binding, mediating transcription of tissue-specific target genes. In contrast, exogenous GC treatment induces GR dimer assembly to classic palindromic site-containing promoters of ligand-dependent genes. This event is at the expense of monomeric GR binding which vanishes from promoters of repressed genes^8^. These finding are important for the improvement of GR-based treatments. Among the widely studied anti-inflammatory actions of GCs is the GR-mediated repression of the activity of pro-inflammatory transcription factors such as NF-κB and AP1 via protein-protein interactions^6^. Although GCs are commonly used in the clinic for their potent anti-inflammatory properties, GCs are not always effective as a treatment due to the onset of glucocorticoid resistance (GCR); a phenomenon observed in many inflammatory conditions^9^.

GR’s post-translational modifications, such as phosphorylation, play a predominant role in determining and fine-tuning the receptor’s function^10, 11^. Accordingly, phosphorylated residues already described to influence the transcriptional activity of human GR are serine 203 (S203) and serine 211 (S211). Phosphorylated GR at S203 (pS203) is cytoplasmically contained and fails to bind GRE-dependent promoters, suggesting that GR pS203 is a transcriptionally inactive form of the GR. However, GR is transcriptionally more active when phosphorylated at S211, due to a conformational change, and an increased recruitment at GRE-containing promoters has been observed^12^.

**Figure 1:**
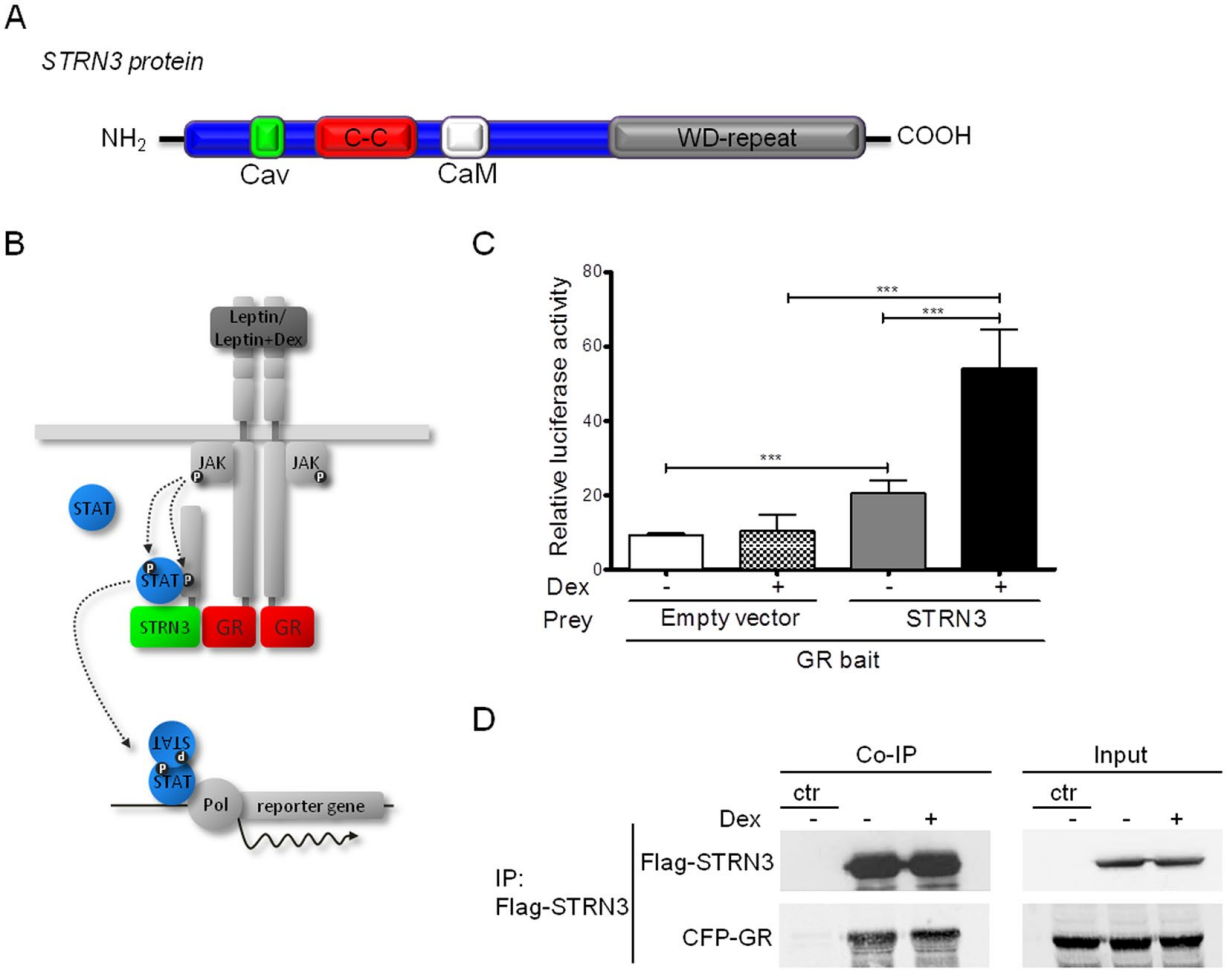
STRN3 is a novel interaction partner of the Glucocorticoid receptor. (**A**) Protein structure of STRN3. Depicted are its representative domains i.e. the Caveolin-binding domain (CaV), the coiled-coil domain (C-C), the Calmodulin-binding domain (CaM) and the tryptophan-aspartate (WD)-repeats at its C-terminal domain. (**B**) Schematic representation of MAPPIT technology. GR is fused to a mutated Leptin receptor, unable to recruit STAT3. The STRN3 pray is bound to a gp130 fragment with functional STAT3 recruitment sites. Only upon interaction of GR with STRN3 and stimulation with Leptin or Leptin + Dex, the Leptin receptor signaling cascade is reconstituted, leading to STAT3 activation, translocation to the nucleus and activation of the reporter gene (luciferase). (**C**) For the MAPPIT experiments, HEK293T cells were transfected with GR bait, rPAP1-Luc reporter and STRN3 prey or empty vector and stimulated with vehicle (medium, not shown in the graph) or leptin plus medium (−) or with leptin plus Dex (+) for 24 h (more details in Methods). The graphs represent the fold change induction to the vehicle-treated condition. Empty vector was used as negative control. The graph represents pool data from three independent experiments. (**D**) The interaction of GR with STRN3 was confirmed with co-immunoprecipitation (Co-IP). HEK293T cells were transfected with 3 μg Flag-STRN3 (96 kDa) and 4 μg of CFP-GR (117 kDa) (or empty vector so that total transfected DNA is 7 μg) and stimulated either with vehicle (media) or with 1 μM Dex for 2 h. Cells were lysed and immunoprecipitated with M2-Flag beads. Over-expression of CFP-GR with M2-Flag beads incubation was used as negative control for the IP assay. Precipitates and input lysates were immunoblotted for GR and Flag. The blots show one representative experiment out of three performed (full-length blots are provided in Supplementary S11).

By using the high-throughput mammalian protein-protein interaction trap (MAPPIT), we identified human Striatin-3 or alternatively S/G2 nuclear autoantigen (SG2NA) isoform alpha, STRN3α (hereafter referred to as STRN3) as a novel interaction partner of GR that negatively affects the GR-dependent transactivation pathway. STRN3 belongs to the striatin family of proteins, which consists of three members, namely Striatin, Striatin-3 and Zinedin, acting mainly as scaffold proteins^13^. The striatin family of proteins associates with kinases as well as phosphatases, including the major eukaryotic serine/threonine protein phosphatase 2 A (PP2A), forming the striatin-interacting phosphatase and kinase (STRIPAK) complex^14, 15^. STRN3 family members share a common protein structure consisting of four well-defined domains i.e. the caveolin-binding domain, the coiled-coil domain, the Ca^2+^-calmodulin-binding domain and the tryptophan-aspartate (WD)-repeat domain^16^ (Fig. 1A). Here, we found that the recruitment of STRN3 to GR indirectly suppresses GR transactivation, by allowing PPP2CA to be additionally recruited to facilitate GR dephosphorylation at S211. Our data thus support a role for STRN3 as an important check-point for GR functionality.

## Results

### STRN3 is a novel interaction partner of GR

**Figure 2:**
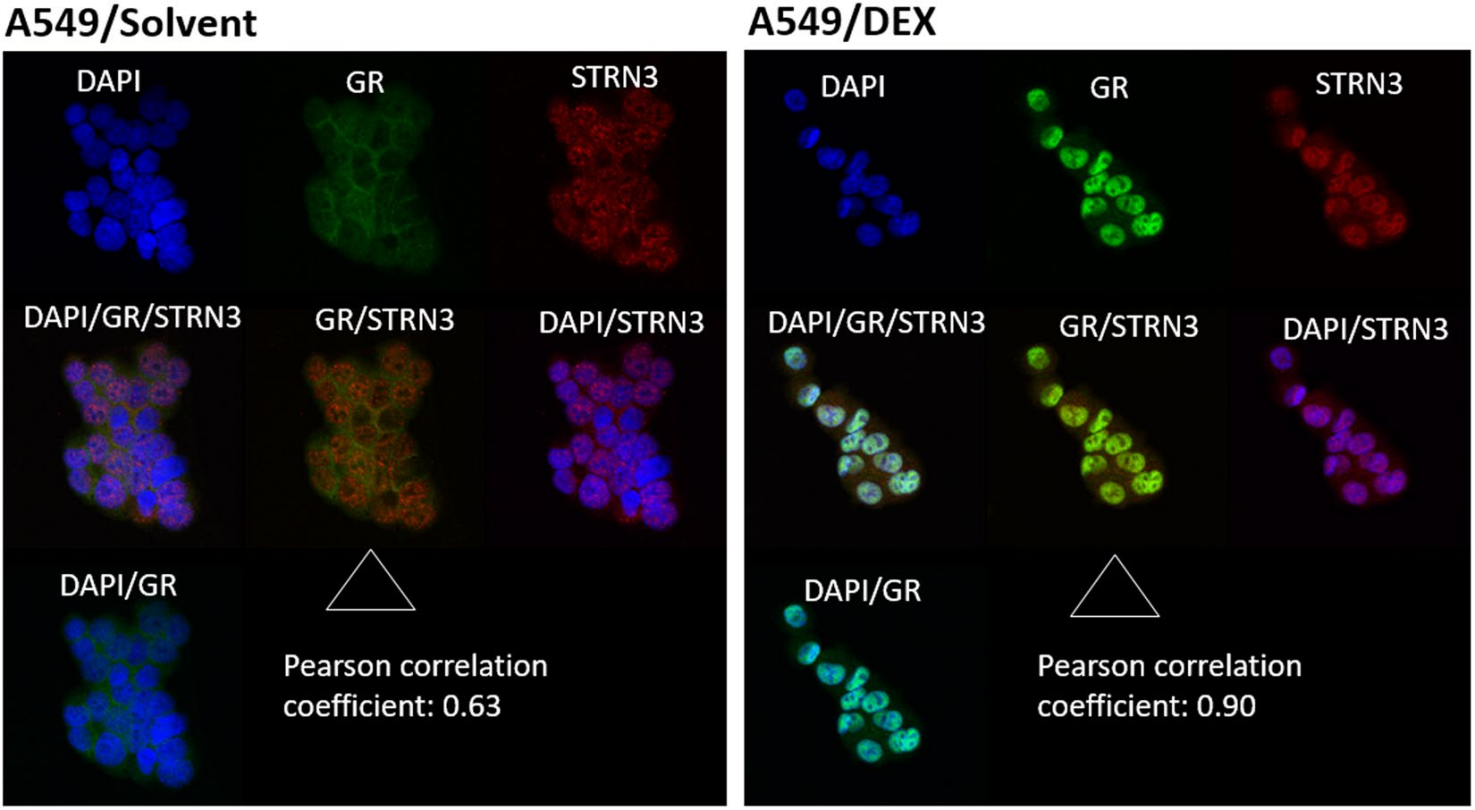
DEX treatment of A549 cells supports co-detection of endogenous GR and STRN3 in the nucleus. A549 cells were seeded on coverslips and incubated in phenol-red free and serum-free medium for 4 h. Cells were treated with either solvent or 1 µM of DEX, for 2 h following by indirect immunofluorescence analysis to detect GR (green signal) and/or STRN3 (red signal). Cell nuclei are visualized using DAPI (blue signal). Assessment of co-localization of the protein signal for GR and STRN3 (middle frame) was performed by correlation statistics using the Olympus Fluoview version 4.2 software. The image is a representative result of 5 recorded images, with each field containing minimally 10 cells.

Array MAPPIT^17^, a two-hybrid technology for the identification and analysis of protein-protein interactions in mammalian cells^18–20^ (Fig. 1B and C), was used to screen for interaction partners of GRα^21^ (hereafter referred to as GR). We identified Striatin-3 (STRN3) (Fig. 1A) as a novel specific interaction partner of GR (Fig. 1C). The interaction between STRN3 and GR was further enhanced upon activating the receptor with the synthetic GR agonist Dexamethasone (Dex) (Fig. 1C). Although the interaction was independently confirmed using co-immunoprecipitation analysis (Fig. 1D), the MAPPIT assay proved more sensitive in detecting a Dex ligand-enhanced interaction. To find out in which subcellular compartment endogenous GR and STRN3 reside, A549 cells were treated with solvent or DEX and subject to indirect immunofluorescence analysis (Fig. 2). In solvent-treated cells, GR was predominantly cytoplasmic, while STRN3 signal was present both in cytoplasm and nucleus. The subcellular distribution changed dramatically upon adding DEX for 2 h. Both GR and STRN3 displayed a predominantly nuclear phenotype, in support of a co-localization. To strengthen the hypothesis, the average Pearson correlation coefficients (PCC), indicative of a co-localization, of whole fields of 5 recorded images of at least 10 cells/field were determined as described by Dunn *et al*.^22^. PCC values range from 1 for two images of which fluorescence intensities are perfectly, linearly related, to −1 for two images of which fluorescence intensities are perfectly, but inversely, related to one another. The average Pearson correlation coefficient of the solvent control set-up for GR and STRN3 was 0.63 while the Pearson correlation coefficient of the DEX set-up for GR and STRN3 increased to a value of 0.90, indicating a linear relation between the signals for GR and STRN3. In conclusion, following DEX activation both proteins are most likely able to interact in the nucleus. In support of a cell type independent effect, similar data were obtained in HeLa cells, which have been reported to contain substantial amounts of endogenous STRN3 (Supplementary S1).

### STRN3 is an inhibitor of the transactivation function of GR

**Figure 3:**
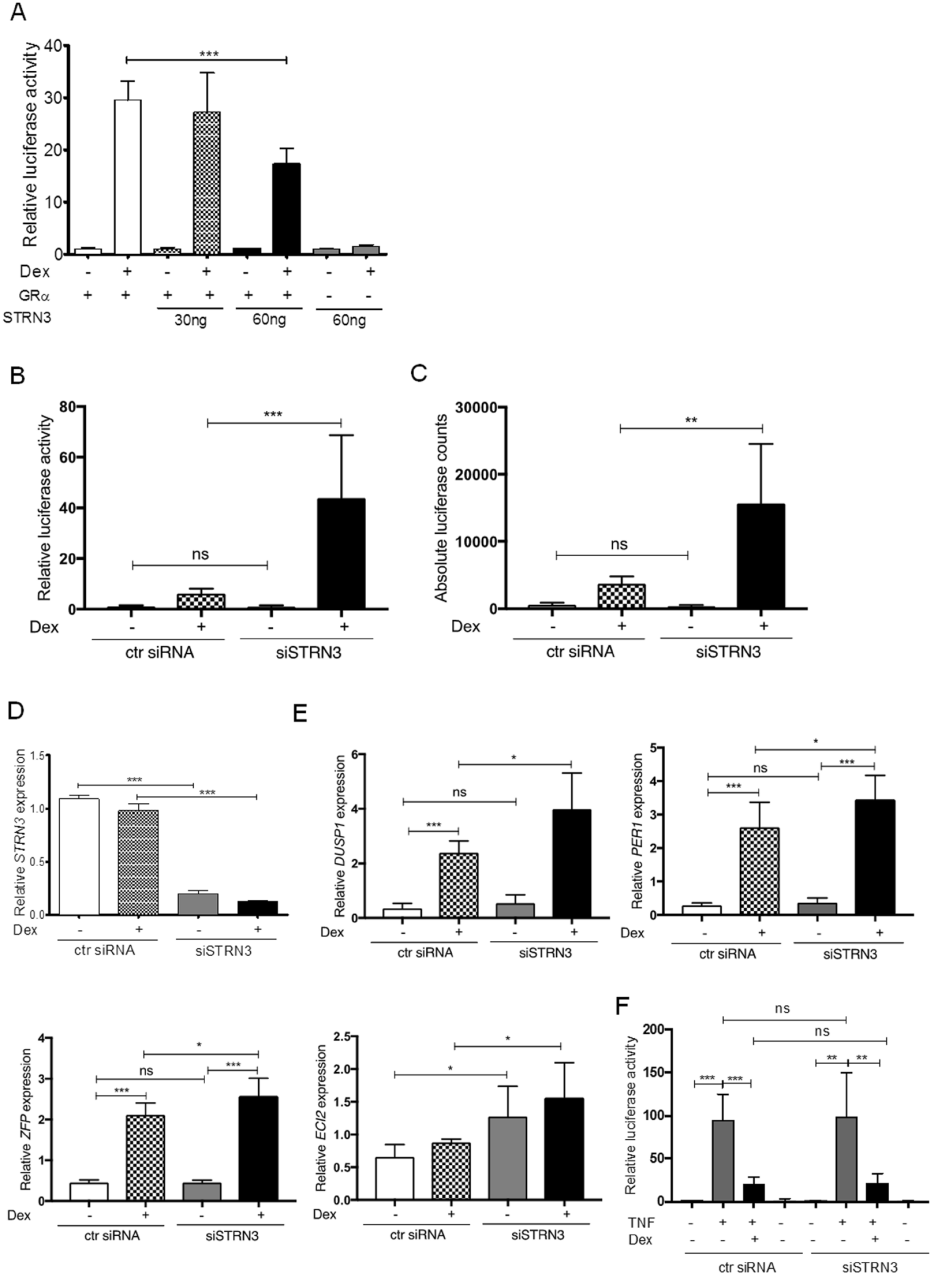
STRN3 is an inhibitor of GR trans-activation. (**A**) HEKT293T cells were transfected in 96-well plate with 30 ng of Flag-GR, 15 ng of GRE-luc and over-expression of STRN3, 30 ng and 60 ng, as indicated (or empty vector so that total transfected DNA is 110 ng). Cells were treated with 1 μM Dex or vehicle, for 6 h. In the last two lanes, Flag-GR was not over-expressed. (**B**) A549 cells stably transfected with GRE-Luc reporter were transfected with 50 nM siRNA On Target plus (Dharmacon) for human STRN3. Cells were treated with 1 μM Dex or vehicle for 24 h and processed for luciferase measurement. (**C**) Representation of the absolute luciferase values of graph in (**B**). (**D**) WT A549 cells were transfected with STRN3-specific siRNA or control siRNA (as in **B** and **C**) and stimulated with 1 μM Dex or vehicle for 6 h before lysis and RNA extraction. A549 cells treated as in (**B**,**C**) were processed for qPCR to determine the endogenous STRN3 levels and (**E**) the levels of the GR-dependent genes DUSP1, PER1, ZFP and ECI2. (**F**) A549 cells stably transfected with NF-κB-Luc reporter are transfected with human STRN3-specific siRNA or control siRNA (as in **B**,**C**,**E**). Cells are subsequently treated with vehicle or with 1000 U/ml of human TNF or pre-treated with 1000 U/ml of human TNF for 1 h followed by 1 μM Dex for 6 h or with 1 μM Dex alone for 6 h. All data are represented as fold change to the vehicle-treated condition. The graphs represent pooled data from three independent experiments.

To test the effect of STRN3 on GR-mediated transcription in mammalian cells, we used both over-expression and silencing approaches. First, a glucocorticoid response element (GRE)-dependent reporter gene construct was transfected in HEK293T cells, with an expression plasmid for GR since these cells endogenously do not contain functional GR^23^. Figure 3A demonstrates that STRN3 can inhibit the Dex-mediated transactivation capacity of GR. The last two control lanes show that STRN3 specifically affects GR-mediated gene expression and does not influence the activity of the reporter in the absence of over-expressed GR. Similar results were observed using HeLa cells, confirming that the effect of STRN3 is cell-type independent (Supplementary S2). To find out whether endogenous STRN3 modulates GR-mediated gene activation, we silenced endogenous STRN3 and monitored both GRE-dependent reporter gene activity (Fig. 3B and C) and endogenous GRE-dependent target genes (Fig. 3E). A549 cells were used, in which we previously optimized the silencing technology^24^ and which advantageously contain endogenous GR. STRN3 knockdown increased the Dex-mediated GRE-dependent reporter gene activity (Fig. 3B). Figure 3C represents the absolute counts of the luciferase activity of Figure 3B. Both Figure 3B and C show that STRN3 silencing does not cause a significant effect on GR basal activity in the absence of Dex. Figure 3D demonstrates an efficient knockdown of STRN3 of over 80%. In line herewith, the Dex-induced GRE-dependent mRNA expression of DUSP1, encoding MKP-1, PER1, ZFP and ECI2 (Fig. 3E), are also enhanced upon silencing of STRN3. Interestingly, the levels of ECI2 are enhanced in a statistical significant manner in the absence of Dex. We believe this result may reflect a promoter specific effect. Moreover, we could confirm the effect of STRN3 silencing on GR transactivation capacity by using a different STRN3 specific siRNA (Supplementary 3).

Activated GR also inhibits gene expression in a DNA-independent manner, via protein-protein interactions; a mechanism referred to as transrepression. To investigate whether endogenous STRN3 may influence transrepression, we studied the impact of STRN3 silencing on the ability of GR to block the activity of NF-κB, using a TNF-induced NF-κB-dependent reporter gene as read-out. Figure 3F demonstrates that STRN3 does not affect the NF-κB-targeting transrepression function of GR. Together; our results indicate that STRN3 may be a specific inhibitor of GR-mediated transactivation, but not transrepression.

### STRN3 inhibits the transactivation function of other nuclear receptors

**Figure 4:**
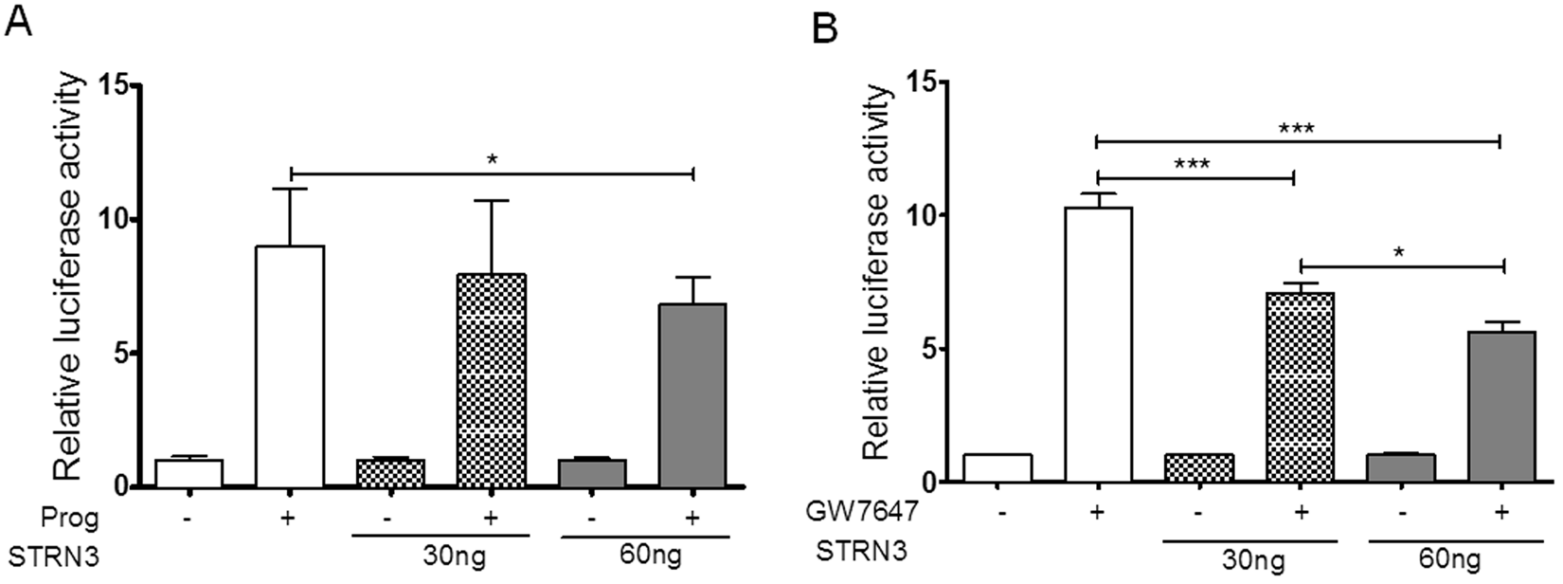
STRN3 inhibits PR and PPARα trans-activation. (**A**) HEK293T cells were transfected with Flag-PR (30 ng) along with its respective PRE-Luc reporter (15 ng) and with 30 ng or 60 ng of Flag-STRN3. The cells were stimulated with 1 μM progesterone (or vehicle) for 24 h as indicated. (**B**) HEK293T cells were transfected with Flag-PPARα (30 ng) along with its respective PPRE-Luc reporter (15 ng) and with 30 ng or 60 ng of Flag-STRN3 (or empty vector so that total transfected DNA is 110 ng). The cells were stimulated with 1 μM GW647 (or vehicle) for 24 h as indicated. The graphs represent pool data from three independent experiments.

As STRN3 was previously described to reduce the activity of ERα^25^, we asked whether the observed effect of STRN3 was specific for the steroid receptor class of nuclear receptors, or else could be expanded to other nuclear receptor family members. Hereto, we compared the effect of STRN3 over-expression on progesterone-driven PR activity (Fig. 4A) and on GW647-driven PPARα activity (Fig. 4B), using their respective promoter elements coupled to luciferase reporters. In analogy with the results using GR, upon over-expression of STRN3 the ligand-mediated transactivation ability of both receptors is decreased. For PR, a decrease of 24% was observed and an even more prominent effect, almost 50%, is obtained for activated PPARα-driven PPRE-Luc activity (Fig. 4B). Already at the lowest concentration of transfected plasmid DNA, STRN3 was able to significantly inhibit PPARα transactivation.

### STRN3 decreases GR transactivation via the dephosphorylation of serine S211

**Figure 5:**
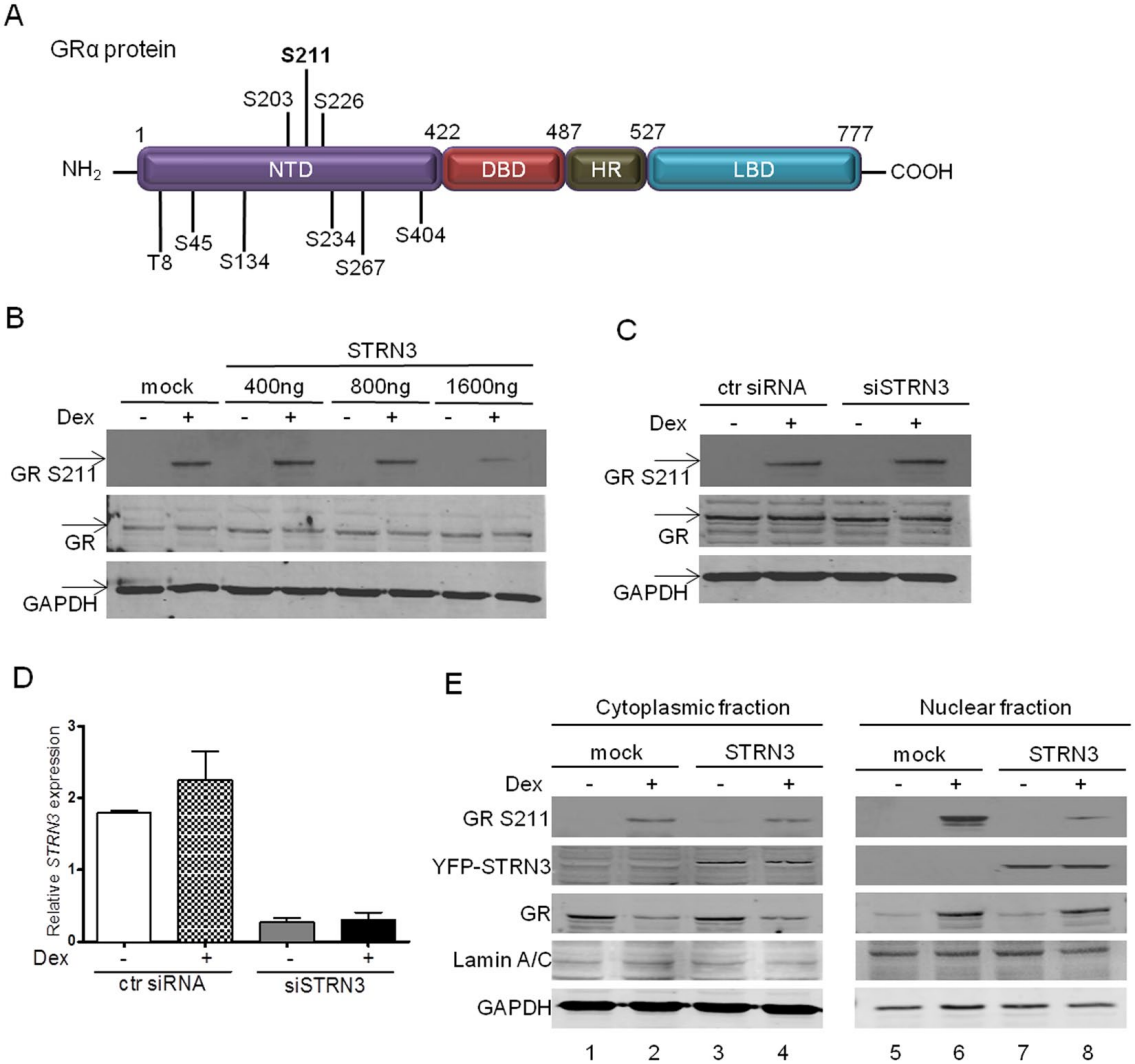
STRN3 mediates the dephosphorylation of GR at serine 211. (**A**) Protein structure of human GR with its respective domains (NTD, DBD, HR, LBD) and the phosphorylation sites indicated (S, serine, T, threonine). (**B**) A549 cells were transfected in 6-wellplate with gradient amounts of YFP-STRN3 plasmid, 400 ng, 800 ng, 1600 ng or mock DNA (empty vector was used so that total transfected DNA is 2 μg/well) and stimulated with vehicle (−) or 1 μM Dex (+) for 2 h. The cells were lysed and processed for immunoblotting against GR phosphorylated at S211 (GR S211), total GR (86 kDa) and GAPDH (36 kDa). (**C**) A549 cells were transfected with 50 nM of STRN3-specific siRNA in 6-wellplate and stimulated with vehicle (−) or with 1 μM Dex (+) for 2 h. Samples were processed either for immunoblotting against GR phosphorylated at S211, total GR and GAPDH (The blots show one representative experiment out of three performed) or for (**D**) RNA isolation and qPCR to determine the endogenous levels of STRN3 and the efficiency of STRN3 silencing (statistics are not performed as data represent a technical triplicate). (**E**) YFP-STRN3 (or mock plasmid, empty vector) was over-expressed in A549 cells at a concentration of 6 μg in 10 cm plates and stimulated with vehicle (−) or with 1 μM Dex (+) for 2 h. Cells were lysed and separated into cytoplasmic and nuclear fractions. Cellular fractions were analyzed by gel electrophoresis and immnunoblotted using antibodies against GR phosphorylated at S211, total GR (86 kDa), (YFP-) STRN3 (122 kDa), Lamin A/C (70 kDa, nuclear marker) and GAPDH (36 kDa, cytoplasmic marker). The blots show one representative experiment out of three performed (full-length blots of 5B, 5C and 5E are provided in Supplementary S12, S13 and S14, respectively).

In human GR, phosphorylation of serine 211 (S211, mouse S220, rat S232) in its N-terminal domain is linked to receptor transactivation^26^ (Fig. 5A). Since we identified a STRN3-mediated decrease of GR transactivation, it was tempting to speculate that STRN3 may alter the phosphorylation status of S211 of human GR. Over-expressing increasing amounts of STRN3 in A549, as indicated, resulted in a gradual decrease in the cellular pool of GR, specifically phosphorylated at S211 upon Dex treatment (Fig. 5B). Of note, the highest transfected STRN3 concentration leads to an 80% repression of S211 phosphorylation (Supplementary S4A).

Complementary herewith, the opposite approach, i.e. silencing of STRN3, demonstrated an increment in the level of GR specifically phosphorylated at S211 upon Dex, of almost 40% (Fig. 5C, Supplementary S4B). Figure 5D demonstrates an efficient knockdown of STRN3. Taken together, these results support that the mechanism by which STRN3 inhibits the transactivation function of GR may be directly linked to receptor dephosphorylation (quantification of the bands is provided in Supplementary S4A and B). A kinetics experiment further showed that the effect of STRN3 on GR phosphorylation is most clear after 90 min Dex (Supplementary S5A and B).

Next, we wanted to test how STRN3 impacts on the nuclear pool of phosphorylated GR, which is able to directly mediate transactivation. Hereto, YFP-STRN3 was over-expressed in A549 cells, and lysates were subjected to a cytoplasmic and nuclear fractionation analysis. In presence of excess STRN3, a decrease in the Dex-mediated GR phosphorylation at S211 is prominent in the nuclear fraction, while no differences on GR distribution between cytoplasmic and nuclear compartments were apparent when comparing the same treatments (Fig. 5E). Hence, the effect of STRN3 on GR phosphorylation does not affect the ability of GR to accumulate in the nucleus, but targets the transactivation function of GR when still present within the nuclear compartment. Because the pS211 GR signal specifically decreases in the nucleus in presence of over-expressed STRN3 (Fig. 5E), we postulate that the interaction in the nucleus is most probably the functionally relevant interaction.

**Figure 6:**
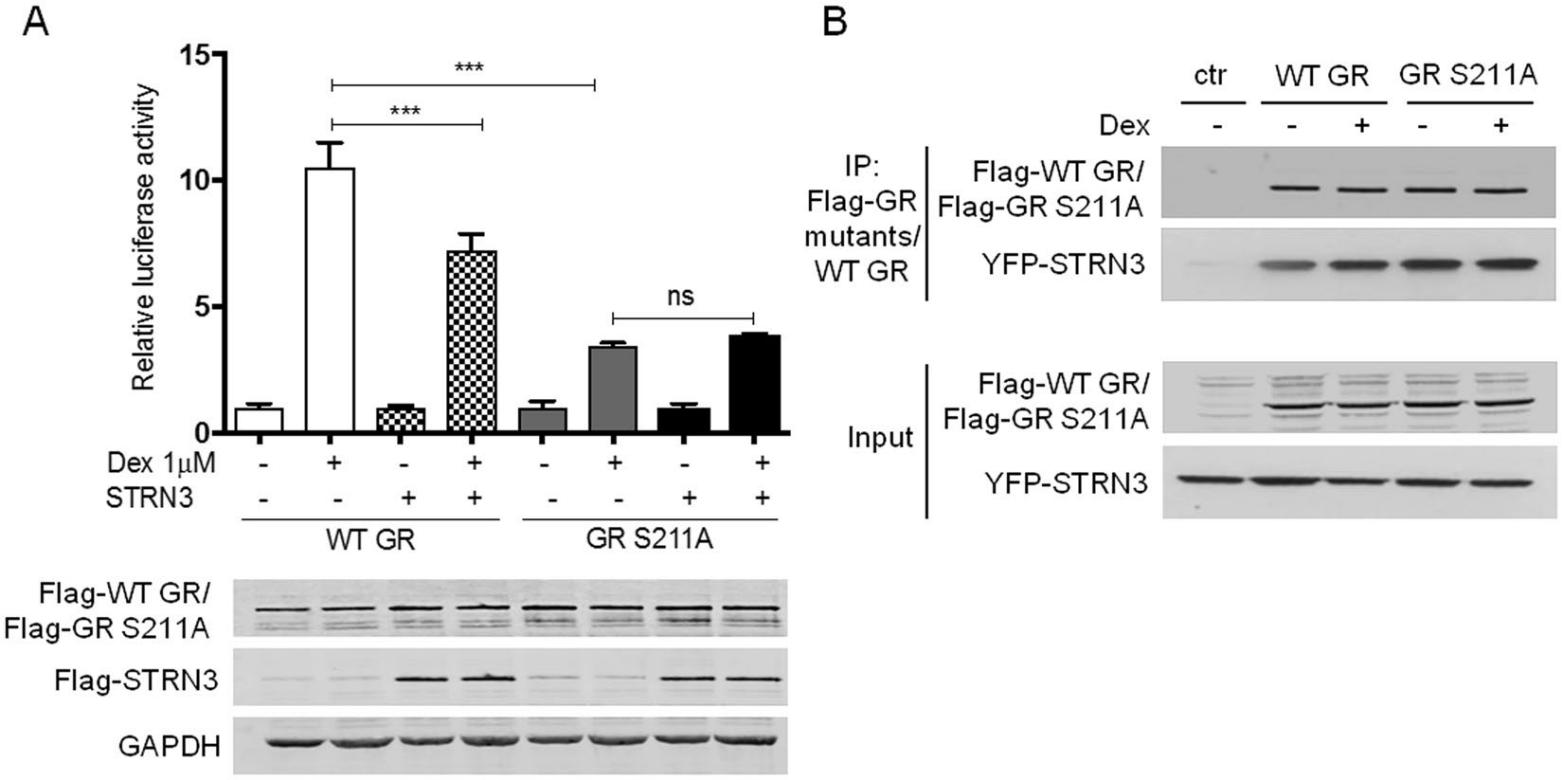
The effect of STRN3 on GR activity is phosphorylation-dependent but not its interaction with GR. (**A**) HEK293T cells were transfected (in 96-wellplate) with 30 ng of WT Flag-GR or with the GR mutant Flag-GR S211A along with 15 ng of the respective GRE-Luc reporter and 60 ng of Flag-STRN3 plasmid where indicated (empty vector was used so that total transfected DNA is 110 ng/well). Stimulation with 1 μM Dex or with vehicle was performed for 24 h. The immunoblots below the graph indicate the transfection efficiency. The graph represents pool data from three independent experiments (**B**) Transfection of HEKT293T cells in 10 cm plates with 3 μg Flag-GR (86 kDa) or Flag-GR S211 A (86 kDa) along with 4 μg of YFP-STRN3 (122 kDa) was performed and stimulation with 1 μM Dex or vehicle. The cells were lysed and processed for co-immunoprecipitation using M2-Flag beads and for immunoblot using anti-Flag and anti-STRN3 antibodies. The experiment was repeated three times with similar results (full-length blots are provided in Supplementary S12).

To strengthen our hypothesis that STRN3 hampers the activity of GR in a phosphorylation-dependent manner, we monitored GR transactivation upon replacing wild-type (WT) GR with a GR variant in which S211 is mutated to Alanine (A), and which is expected to have a lower, yet residual, activity compared to the WT GR^27^. STRN3 has now lost the ability to inhibit GR transactivation (Fig. 6A), confirming our earlier data that STRN3 mediates its effect by targeting phosphorylated GR at S211. To confirm that this mutation does not affect the interaction, which may in essence lead to the same conclusion, again a co-immunoprecipitation was performed. The result demonstrates that Flag-GR S211A is still able to physically interact with YFP-STRN3 (Fig. 6B).

### PPP2CA mediates the effects of STRN3 on GR phosphorylation

**Figure 7:**
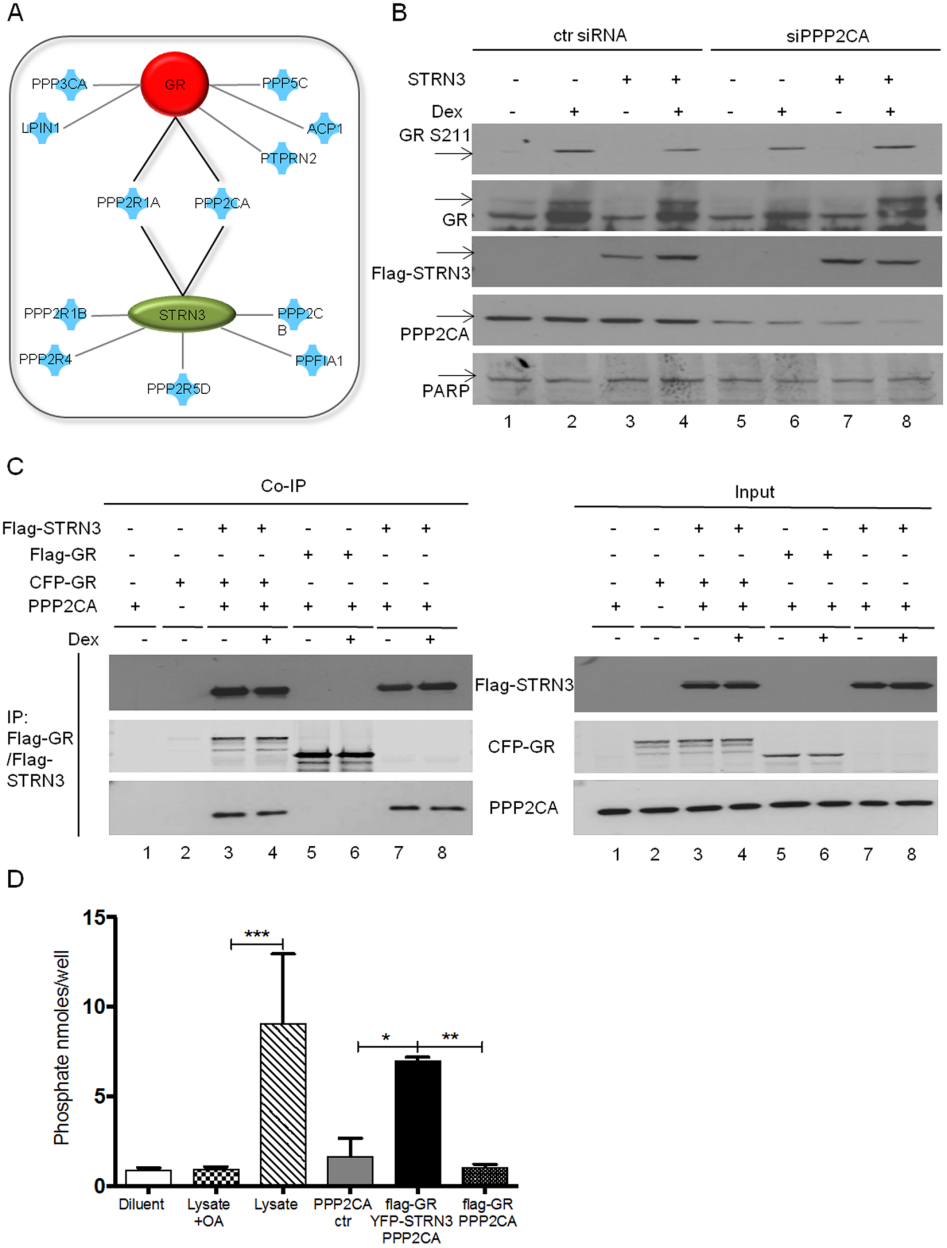
PPP2CA mediates the effect of STRN3 on GR phosphorylation. (**A**) Identification of common interacting phosphatases for STRN3 and GR using Ingenuity Pathway Analysis (IPA) database. (**B**) A549 cells were transfected in 10 cm plates with 50 nM of control (lanes 1–4) or with PPP2CA (lanes 5–8) siRNA. After 24 h, Flag-STRN3 (lanes 3–4, 7–8) or mock (lanes 1–2, 5–6) plasmids were over-expressed (6 μ of total DNA was transfected per plate). After 48 h, the cells were treated with vehicle or 1 μM Dex for 2 h and lysed. The nuclear fractions of the lysates were analyzed by immunoblotting against GR phosphorylated at S211, total GR (86 kDa), (Flag-) STRN3 (96 kDa), PPP2CA (35 kDa) and PARP (116 kDa, nuclear marker). Total GR expression was identified in a separate immunoblot to avoid multiple stripping of the membrane. This is a representative experiment out of three independent experiments that have been performed with similar results. (**C**) Co-immunoprecipitation experiment in HEK293T cells for the complexes GR/STRN3/PPP2CA, GR/PPP2CA and STRN3/PPP2CA (in 10 cm plates). Over-expression of 4 μg PPP2CA (lane 1) and over-expression of 4 μg CFP-GR (lane 2) serve as negative controls. Additionally, the cells were transfected with 3 μg Flag-STRN3, 4 μg CFP-GR and 4 μg PPP2CA (lanes 3–4) or with 3 μg Flag-GR and 4 μg PPP2CA (lanes 5–6) or with 3 μg Flag-STRN3 and 4 μg PPP2CA (lanes 7–8). 48 h after transfection, the cells were treated with vehicle or with 1 μM Dex as indicated (2 h). The lysates were used for IP assays using M2-Flag beads and the precipitates were analyzed with immunoblotting against (Flag-) STRN3 (96 kDa), (CFP- or Flag-) GR (117 or 86 kDa, respectively) and PPP2CA (35 kDa) (11 μg of total DNA was transfected per plate) (full-length blots of (**B,C**) are provided in Supplementary S16 and S17, respectively). (**D**) The complexes Flag-GR/YFP-STRN3/PPP2CA and Flag-GR/PPP2CA were examined for their phosphatase activity. HEK293T cells (in 10 cm plates) were transfected with 4 μg PPP2CA (negative control) or with 3 μg Flag-GR, 4 μg YFP-STRN3 and 4 μg PPP2CA or with 3 μg Flag-GR and 4 μg PPP2CA. All the samples were treated with 1 μM Dex for 2 h. The complexes were precipitated with M2-Flag beads and then incubated with Flag peptide in order to detach the complexes from the beads. Then the samples were processed for the phosphatase assay (the assay is described in the section of Methods). Additional negative controls: no-substrate condition (diluent) and total lysate from cells stimulated with 100 nM Okadaic acid, a phosphatase inhibitor. Positive control: total cell ﻿lysate (11 μg of total DNA was transfected per plate). B, C and D are representations of a series of triplicate experiments that all yielded similar results.

STRN3 is a scaffold protein without enzymatic activity yet interacts with phosphatases and kinases. More specifically, STRN3 can bind to different subunits of protein phosphatase 2 A (PP2A) such as PPP2R1B, PPP2R1A, PPP2CB and PPP2CA^16, 28^. Ingenuity pathway analysis (IPA) was used to identify common phosphatases shown to bind both STRN3 and GR. This yielded two common phosphatases, namely, PPP2R1A and PPP2CA (Fig. 7A). Should one of these phosphatases be directly involved, specific silencing would maintain levels of phosphorylated S211 upon Dex despite the presence of STRN3. Following an efficient knockdown of PPP2CA protein of over 60% (Fig. 7B lanes 5–8, Supplementary S6), this prediction is exactly the result as observed in Fig. 6B. Upon siRNA targeting of PPP2R1A, S211 phosphorylation levels of GR were not affected (data not shown).

Having found that PPP2CA mediates the effects of STRN3, we next reasoned that the three proteins might co-exist in the same protein complex. Co-immunoprecipitation analysis following over-expression of all three components in HEK293T (Fig. 7C) demonstrates that GR, PPP2CA and STRN3 may be part of one complex, potentially explaining effects on GR phosphorylation and subsequently on transactivation. In line with our previous results, using co-immunoprecipitation to study the interaction between GR and STRN3 (Figs 1D and 6B), no ligand effect of Dex on the formation of the trimeric complex is observed. As expected, we observe a strong interaction between PPP2CA and STRN3 conform literature^29^. Surprisingly, we do not detect a previously described interaction between over-expressed GR and PPP2CA^30^. Our results indicate STRN3 as a crucial scaffold protein facilitating the interaction between GR and PPP2CA, which serves to modulate the function of GR. Since endogenous STRN3 could not be detected due to poor antibody quality specifically for IP and Western analysis, we checked in A549 cell lysate whether over-expressed and detectable Flag-STRN3 could pull down endogenous phosphorylated GR and/or endogenous PPP2CA. The result in Supplementary S7 demonstrates that upon immunoprecipitation of Flag-STRN3 endogenous GR phosphorylated at S211 was enriched already in absence of Dex. Although in presence of Dex, the co-immunoprecipitated pS211GR signal is similar, we speculate that this result nevertheless reflects an ongoing dephosphorylation, since in the input the ratio of pS211 GR for Dex-treated cells compared to solvent-treated cells is much higher. Co-immunoprecipitation analysis also demonstrated an additional interaction with endogenous PPP2CA, which again points to the presence of all three proteins in one complex.

To validate the importance of STRN3 for the recruitment of PPP2CA in the same complex with GR, we applied a phosphatase activity assay to compare the levels of phosphatase activity in the complexes in absence and presence of STRN3. Figure 7D shows that a GR-associated phosphatase activity is only detectable when Flag-GR, YFP-STRN3 and PPP2CA are over-expressed, confirming that STRN3 may be prerequisite for the recruitment of PPP2CA in the proximity of GR, whilst in the absence of STRN3, PPP2CA is not able to bind to GR.

### The C-terminal domain of GR is important for a proper formation of the trimeric complex

**Figure 8:**
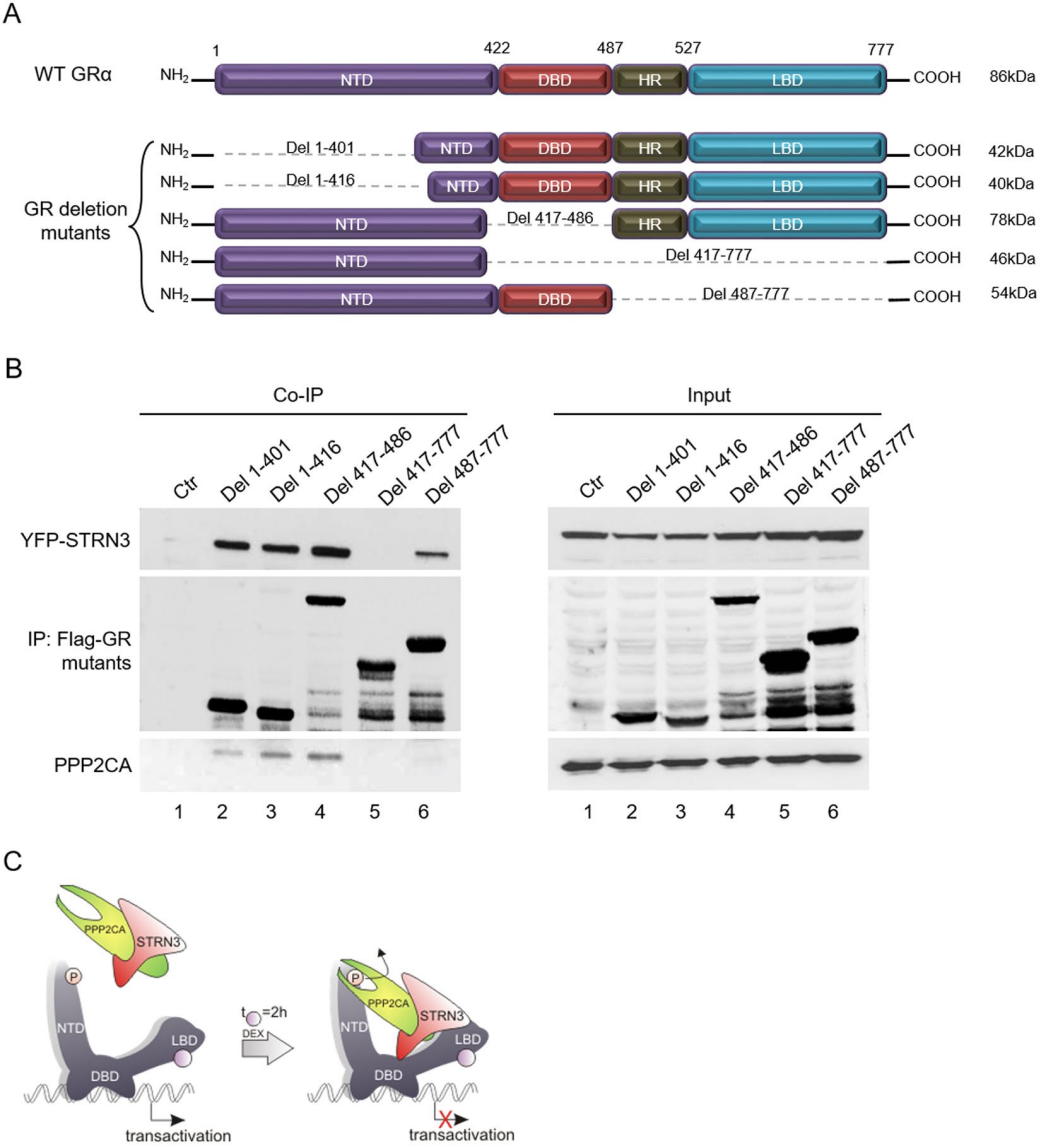
The formation of the trimeric complex GR/STRN3/PPP2CA is dependent on the ligand-binding domain of GR. (**A**) Representation of the GR deletion mutants that were used for mapping GR domains important for the formation of the trimeric complex. (**B**) HEK293T cells were transfected in 10 cm plates with 3 μg of the different Flag-GR deletion mutants along with 4 μg of YFP-STRN3 (122 kDa). 48 h post-transfection the cells were lysed and processed for co-immunoprecipitation with M2-Flag beads (7 μg of total DNA was transfected per plate). The resulting complexes were analyzed with gel electrophoresis and immunoblotting using antibodies against Flag, STRN3 and PPP2CA. In this experiment PPP2CA was not over-expressed and we rely on the endogenous PPP2CA binding to the complex. This is a representation of a series of triplicate experiments that all yielded similar results (full-length blots are provided in Supplementary S18). (**C**) Proposed model for the formation of the trimeric GR-STRN3-PPP2CA complex. GR LBD (and to a lesser extent GR DBD) binds STRN3, allowing PPP2CA to be recruited and to dephosphorylate GR, leading to hampered transactivation. GR with its protein domains (NTD, DBD, LBD) is depicted in grey. P stands for phosphorylated S211. The purple circle represents the ligand, Dex.

We used various Flag-tagged GR deletion mutants encompassing different functional domains (Fig. 8A) of human GR to identify which region(s) is/are important for the interaction with STRN3 and PPP2CA (Fig. 8B). GR mutants carrying a deletion in the NTD or DBD, were all found to strongly interact with YFP-STRN3 (Del1-401, Del1-416, Del417-486). Actually, in all deletion variants of GR in which LBD is still present, a strong interaction with STRN3 is observed, suggesting an important role for this domain of GR. However, the interaction of GR with STRN3 is not lost, only weaker, when the GR-LBD is deleted (Del487-777). This result points to a role for (an)other domain(s) besides the LBD. Yet, if only the N-terminal of the GR is withheld, then the interaction is lost (Del417-777), dismissing this domain as an important contact point. Since a weak interaction is restored using GR Del487-777, in which both NTD and DBD are present, we could envisage a weak contact point involving the DBD. Contrary to this assumption, the deletion mutant Del417-486, which keeps only NTD and LBD intact, exhibits a strong interaction. However, bearing in mind LBD is still present in this mutant, it may be that interaction surfaces are more favorable between GR LBD and STRN3 for this particular GR mutant lacking only DBD. At any rate, domain analysis might not reflect proper folding and so misleading conclusions could be made. Nevertheless, our observations consistently support that the LBD of GR is important for this interaction, even though the LBD alone may not be sufficient for an optimal interaction.

We next studied which GR mutants allow for an additional association of PPP2CA and, hence, trimeric complex formation. As HEK293T cells express high levels of endogenous PPP2CA, no over-expression was needed to detect the binding of endogenous PPP2CA to YFP-STRN3 and Flag-GR deletion mutants. GR LBD is a prerequisite for the binding of PPP2CA to the trimeric complex since in both experimental set-ups using GR minus its LBD (Del487-777 and Del417-777), PPP2CA binding is completely absent (Fig. 8B). Even in the set-up using GR Del487-777, which still allows for a weak interaction with STRN3, the absence of GR LBD prevents a physical recruitment of PPP2CA to the complex, emphasizing again the importance of GR LBD. PPP2CA binds to the coiled-coil domain of STRN3, located close to the N-terminal domain of the STRN3 protein^31^. Consequently, we hypothesize that the LBD of GR interacts with STRN3 so that the coiled-coil domain of STRN3 can bring PPP2CA physically to GR LBD, from where it can reach its target, i.e. the N-terminally localized phosphorylated S211. In conclusion, our results suggest multiple contact points between GR and STRN3 and a prerequisite for GR LBD to enable a functional trimeric complex with PPP2CA, leading to a model as depicted in Fig. 8C.

## Discussion

Because of the role of GR in different biological pathways and its use as a drug target^32^, analysis of its interactome contributes to comprehend its regulatory pathways. Here, STRN3 was identified as a novel interaction partner of liganded GR. Immunofluorescence analysis demonstrated that these two endogenous proteins co-localize in the nucleus, indicating the involvement of STRN3 in GR’s nuclear actions.

Over-expression of STRN3 decreased GR transactivation while silencing enhanced GR transactivation. In line herewith, STRN3 silencing also augmented endogenous mRNA levels of the GR-inducible target genes DUSP1, encoding MKP1, a potent anti-inflammatory mediator^33^, PER1, ZFP and ECI2. Of note, the levels of ECI2 were enhanced upon silencing of STRN3 even in the absence of Dex treatment. Since ECI2 is a PPARα/GRα co-controlled gene^34^ and PPARα may be also a target of STRN3 (Fig. 4B) it is tempting to speculate that the different regulation of ECI2 might be ascribed to an increased level control of this particular gene promoter. Interestingly, silencing of STRN3 did not interfere with the trans-repression pathway of GR (Fig. 3E) indicating that STRN3 may influence only the dimerization-dependent actions of GR. Phosphorylation of GR at S211 is a known prerequisite for the transcriptional activity of the receptor^10, 27, 35^. Over-expression of STRN3 causes decreased phosphorylation of GR at S211, whilst silencing of STRN3 leads to the enhancement of GR phosphorylation at the same residue. By using a GR mutant of S211 (S211A) we showed that the negative impact of STRN3 on GR transactivation is phosphorylation-dependent. Since the striatin family of proteins lacks enzymatic activity, a phosphatase may be recruited within the same complex that can alter the phosphorylation status of GR in the presence of STRN3. Tan and colleagues described a negative effect of rat STRN3γ on the actions of human Estrogen receptor α through dephosphorylation of the receptor at S118 by the catalytic subunit of PP2A, PPP2CA^25^. Although according to IPA, PPP2R1A could also be a candidate, in accord with the study of Tan *et al*. on ERα, only PPP2CA could be identified as the responsible enzyme dephosphorylating GR upon STRN3 over-expression. Its silencing lifts the negative effect of STRN3 on GR’s phosphorylation status, which further strengthens the hypothesis of a trimeric complex. Following amino acid sequence alignment analysis of human ERα with human GR, we observe that S118 of ERα does not correspond to S211 of GR (Supplementary S8), indicating a different mechanism of action by which STRN3 and PPP2CA control the activity of these two different, yet related, receptors. Our results indicate that STRN3 may act as an inhibitor not only of steroid but also of peroxisome proliferator-activated nuclear receptors. Of note, STRN3 has been described in the literature to modulate the actions of ER, as mentioned above, but also of the Mineralocorticoid receptor (MR)^36^. Apart from the nuclear effect of STRN3 on ER, it is also involved in the receptor’s membrane associated actions. More specifically, over-expression of striatin in endothelial cells resulted in an increase in ERα in the membrane-enriched fraction, containing EGFR and IGF-1 receptor (IGF-1R), and a slight decrease in nuclear ERα. Striatin serves as a scaffold directing ERα to the plasma membrane and bridges ERα with the GPCR (Gαi) complex to facilitate assembly of a membrane signaling complex required for rapid estrogen extra-nuclear activation of MAPK, Akt, and eNOS in endothelial cells^37–39^. A similar mechanism has been proposed for Striatin and MR. Accordingly, in endothelial cells and murine heart tissue, complexes between striatin and MR have been described that can be disrupted by aldosterone but cannot be restored by spironolactone^40, 41^. Activation of MR by high levels of aldosterone increases striatin levels in vascular cells and in tissues of mouse models with elevated aldosterone concentrations^40, 42^. Lowering striatin levels in endothelial cells reduces the aldosterone-mediated non-genomic MR-dependent ERK phosphorylation without affecting EGF-induced ERK phosphorylation or genomic MR signaling^43^. The relevance of striatin for MR signaling is further suggested by the analysis of heterozygous striatin KO mice with low striatin levels and salt sensitive blood pressure. In this model, the pAKT/AKT ratio, as part of another potential non-genomic MR signaling pathway, is reduced while MR expression and genomic signaling are increased^44^. Collectively, our data reporting the effect of STRN3 on GR and the first evidences for a possible effect on the nuclear actions of PR and PPARα, together with the published findings by other research groups on other steroid receptor members, pinpoint the STRN family of proteins as a prominent regulator of nuclear receptor actions.

Co-immunoprecipitation analysis revealed the possibility of the formation of a trimeric complex among GR, STRN3 and PPP2CA, however, with the present data we cannot conclude whether these interactions are direct, and/or whether more proteins are involved. In the absence of STRN3 over-expression, GR was not able to interact with PPP2CA in HEK293T. However, the interaction of PPP2CA with GR has been described previously to contribute to corticosteroid sensitivity in severe asthma by dephosphorylation of GR S226, a modification regulating receptor nuclear export^30, 45^. In our study, over-expression of STRN3 and the subsequent recruitment of PPP2CA did not affect receptor shuttling. The discrepancy between results may be attributed to the different cell lines tested. We believe that PPP2CA needs an intermediate protein to form the complex in which GR is recruited. Here, STRN3 serves as the scaffold protein that will attract PPP2CA to a complex with GR. Nevertheless, in a different cell line and under different conditions, PPP2CA might use another scaffold protein to engage GR in the complex. Another example in which STRN3 is an essential part of a trimeric complex is with the antioxidant protein DJ-1 and the survival kinase Akt, suggestive of a role in neuroprotection^46^. Combining the co-immunoprecipitation data for (phosphorylated) GR with the immunofluorescence-based endogenous co-detection between GR and STRN3, we bring forward that the formation of a protein complex between ligand-activated GR, STRN3 and PPP2CA most likely occurs in the nucleus, hereby influencing the transactivation capacity of GR.

Co-immunoprecipitation experiments with GR deletion mutants indicated that predominantly the LBD and, to a lesser extent, the very C-terminal part of the NTD spanning into the DBD of the GR are needed to interact with STRN3 while the LBD is crucial for the binding of endogenous PPP2CA. STRN3 oligomerizes and interacts with PPP2CA via its coiled-coil domain^31^. Collectively, we deduct that GR and STRN3 might interact in an antiparallel orientation so that the C-terminal domain of GR is in close proximity with the coiled-coil domain of STRN3, which are also the respective domains for PPP2CA binding.

Apart from the well-studied role of the GR-mediated transrepression pathway in immunosuppression^47, 48^, the transactivation pathway of GR has re-gained attention due to the activation of a cluster of genes with potent anti-inflammatory actions such as GILZ and DUSP1^49^. One of the causes leading to GCR, a state in which the patients do not respond to the GC treatment^50^, is the elevated levels of Tumor Necrosis Factor (TNF); a potent pro-inflammatory cytokine^33^. TNF and GR interfere with each other’s signaling pathway, resulting in a mutual inhibition^51^. As proven before *in vitro* and *in vivo* treatment with Dex followed by TNF, diminished the efficiency of Dex and the subsequent GR activity by almost 50%, (Supplementary S9)^52, 53^. Preliminary data indicate that in liver samples from wild type mice injected with TNF, endogenous mRNA levels of STRN3 were induced upon TNF treatment (Supplementary S10A). It would be interesting to further investigate whether in acute inflammatory conditions induced by TNF, the inflammatory environment may alter the interactome of GR, favoring the up-regulation of co-factors with a potential negative effect on GR functionality, as a possible mechanism leading to GCR (Supplementary S10B). In conclusion, STRN3, the novel inhibitor interaction partner of GR, may contribute to a continuous recruitment of PPP2CA at the GR-STRN3 complex followed by a dephosphorylation of GR at S211, resulting in a decrease of GR transactivation (Fig. 8C). The mechanism may involve a platform created by GR and STRN3, which further assists PPP2CA to anchor to the complex and to mediate the dephosphorylation of GR at S211, linking to an overall diminished transactivation function of GR.

## Methods

### Plasmids

The MAPPIT pCLG-hGRα bait vector was generated by cloning the full size human GRα coding sequence in the pCLG vector backbone, which was described previously^54^. The pMG1-hSTRN3 prey vector was obtained by Gateway recombinatorial transfer of a full size STRN3 ORF cDNA from an entry clone selected from the human ORFeome collection version 5.1^55^ into the pMG1 destination vector, as reported before^56^. A STAT3-responsive firefly reporter plasmid, pXP2d2-rPAP1-Luc^57^, was used to generate the MAPPIT readout.

The functional reporter assays involved the following plasmids: GRE-Luc reporter plasmid, which contains two copies of the glucocorticoid response element (GRE) and the pHD-Luc that contains the PPARα response element^58^. The human STRN3a from the human ORFeome V5.1 collection was cloned in two different vectors, pMet7-Flag-STRN3 and pMet7-YFP-STRN3a. hGRα and hPPARα were cloned into a pMet7-Flag vector, a pECFP-GRα and a pMet7-Flag-PPARα, respectively. The GR mutant with serine 211 (S211) mutated into Alanine (GR S211A) was generated via site directed mutagenesis of pEF-Flag-human GRα and checked by sequencing analysis. The primers used in PCR containing the mutation are the following: CCCCAGGTAAAGAGACGAATGAGGCTCCATGGAGATCAGACCTGTTG and CAACAGGTCTGATCTCCATGGAGCCTCATTCGTCTCTTTACCTGGGG. GR deletion mutants were cloned in pMet7-Flag and were chemically synthesized by Gene9 containing att sites for gateway cloning in the destination vectors. The mutants span the following deletions: Del1-401, Del1-416, Del417-486, Del417-777 and Del487-777. The human PPP2CA plasmid is from addgene and is cloned into a pBABE zeo vector^59^. pMet7-Flag-empty vector was used as mock DNA where appropriate, to reach the same amount of transfected DNA per well for all set-ups.

### Antibodies

Anti-GR (rabbit, H300, Santa cruz Biotechnology), anti-PPP2CA (rabbit, Proteintech), anti-Phosphorylated S211 Glucocorticoid receptor (rabbit, cell signaling technology), anti-STRN3 [1) N-20, goat polyclonal, sc:16853, Santa cruz Biotechnology, used in immunofluorescence experiments, 2) S68, mouse monoclonal, MAI-46461, Thermo Scientific, 3) mouse monoclonal, 05-1115, Millipore] (1 and 2 successfully detect over-expressed STRN3 in western blots), anti-Flag (mouse, Sigma), anti-Flag (rabbit, Sigma), anti-Lamin A/C (mouse, Cell signaling technology), anti-GAPDH (rabbit, Abcam), anti-PARP-1 (H-250, Santa cruz Biotechnology), horseradish peroxidase-conjugated anti-mouse and anti-rabbit from Jackson Immunoresearch laboratories, Pierce donkey anti-rabbit secondary antibody dylight 800 or 680 and Pierce donkey anti-mouse secondary antibody dylight 680 (Thermo Scientific).

### Cell culture

Human embryonic kidney T cells (HEK293T) and human lung carcinoma (A549) cells, basal or stably transfected with recombinant NF-κB- or GRE-dependent reporter genes^60^ were maintained in DMEM plus fetal bovine serum in a humidified atmosphere of 5% CO_2_ at 37 °C.

### MAPPIT

HEK293T cells were cultured in 96-well microtiter plates in DMEM supplemented with 10% fetal bovine serum and transfected with bait (25 ng per well), prey (50 ng per well) and reporter (5 ng per well) plasmids, via standard calcium phosphate transfection, as described earlier^56^. Twenty-four hours after transfection, triplicate wells were treated either with vehicle (medium, non-active MAPPIT system) or leptin (100 ng/ml) plus medium or leptin plus Dex (1 µM). Another 24 h later, luciferase activity was measured using the Luciferase Assay System kit (Promega) on an Envision plate reader (Perkin Elmer)^21^. Luciferase fold change was determined by calculating the ratio of either leptin + medium/vehicle or leptin + Dex/vehicle (Fig. 1C). The amount of DNA transfected per well was kept constant to 100 ng/well by addition of empty vector plasmid.

### Indirect Immunofluoresecence analysis

Cell fixation, methanol permeabilization and staining of A549 or HeLa cells were performed according to Cell Signaling guidelines. GR was visualized with the GR polyclonal (H300) antibody (Santa Cruz) while STRN3 was visualized with the STRN3 monoclonal (N20) antibody (Santa Cruz), both used at 1:200, followed by probing with Alexa Fluor® 488 (Invitrogen) and/or Alexa Fluor® 568 (Invitrogen), respectively at a concentration of 1:1000. Nuclei were visualized using 4′,6-diamidino-2-phenylindole (DAPI) staining at a concentration of 1:100. A motorized inverted IX81 FluoView FV1000 laser scanning confocal microscope (Olympus) was used to record high-resolution images.

### Reporter gene assay

HEK293T cells were transfected with calcium phosphate. Plasmids encoding GR, PR and PPARα along with their respective response element-dependent reporters, GRE-Luc, PRE-Luc and PPRE-Luc were over-expressed in presence of a constitutive β-galactosidase expressing plasmid and in absence or presence of increasing amounts of Flag-STRN3 plasmid, as indicated in ﻿Figures’ legends﻿. The experiments were performed in 96 well plates. 24 h after transfection, the medium was replaced with Optimem and 48 h after transfection the cells were stimulated with the respective nuclear receptor ligands for 6 h. Firefly luciferase read-outs were normalized to β-galactosidase activity.

### Small interfering RNA (siRNA) transfection

All siRNAs used in this study were purchased from Dharmacon. Two different siRNA specific for human STRN3 were used to confirm the specificity of the assay [siGenome Smartpool (M-019145-01-0005) and On-target plus Smartpool (L-019145-01-0005)]. For human PPP2CA silencing, we used the On-target plus Smartpool siRNA (L-003598-01-0005). As a control siRNA, siControl Nontargeting was used. A549 cells were transfected with a final concentration of 50 nM with the above-mentioned siRNAs with Dharmafect 1 (Thermo Scientific) reagent according to the manufacturer’s instructions. Using reporter gene assays, 72 h after siRNA transfection, the cells were treated as specified under “Results”. In case of an additional plasmid transfection step, 24 h after siRNA transfection, the medium was changed to Optimem followed by plasmid DNA transfection of A549 cells using the Lipofectamine Plus protocol according to the manufacturer’s instructions. 48 h later, the cells were further processed for analysis.

### Quantitative RT-PCR (qPCR)

Total RNA was prepared from A549 cells or mouse liver samples using the RNeasy mini kit (Qiagen). cDNA was synthesized using iScript^TM^ Advanced cDNA Synthesis Kit from Bio-Rad and analyzed using the Power SYBR Green Master Mix (Applied Biosystems). Expression levels were calculated using the comparative C_t_ method, normalized to the best performing housekeeping genes, GAPDH and β-actin, determined by Genorm^61^.

### Co-immunoprecipitation and Western analysis

Experiments were performed in 10 cm petri dishes. HEK293T cells were harvested and homogenized in lysis buffer A (10 mM Hepes pH 7.5, 1.5 mM MgCl_2_, 10 mM KCL, 0.5 mM DTT, 0.1% NP-40 supplemented with a protease inhibitor cocktail (Roche)). Samples were subjected to two freeze-thaw cycli (−70 °C). Lysates were cleared by centrifugation at 13.000 rpm at 4 °C and incubated with 20 μl of Anti-Flag M2 Affinity Gel (Sigma Aldrich) overnight at 4 °C. Beads are washed 4× in buffer A supplemented with 150 mM NaCl and 0.5% Triton-X 100, re-suspended in Laemmli buffer and boiled for 1 min at 95 °C. Immunoprecipitates were either frozen at −20 °C or used for Western analysis using antibodies (diluted in blocking buffer at 1/1000 overnight) against GR, STRN3, Flag and PPP2CA.

### Nuclear and cytoplasmic fractionation

Cells were collected and resuspended in 200 μl of ice-cold Buffer 1 pH 7.5 (10 mM Hepes, 10 mM KCL, 1 mM MgCl_2_, 5% glycerol, 0.5% EDTA, 0.5% EGTA) supplemented with protease inhibitor (Roche) and incubated on ice for 15 min. 1 μl 10% NP-40 was added prior to a vortexing step for 10 sec. The cytoplasmic fraction was collected after centrifugation for 5 min at 14.000 rpm at 4 °C. To the remaining pellet, 100 μl of Buffer 2 pH 7.5 (10 mM Hepes, 1% NP-40, 1 mM MgCl_2_, 400 mM NaCl, 10 mM KCl, 20% glycerol, 0.5 mM EDTA, 0.5 mM EGTA) was added, followed by a vortexing step for 2 sec and incubation at 4 °C for 30 min on a shaker. The nuclear fraction was collected after centrifugation for 15 min at 14.000 rpm at 4 °C.

### Phosphatase activity assay

The DuoSet^®^ IC kit, specific for the quantification of human/mouse/rat PP2A activity (R&D systems), was used according to the manufacturer’s instructions. This DuoSet^®^ IC activity assay contains the basic components required for the development of capture assays to measure the activity of PPA in lysates. An immobilized capture antibody specific for the catalytic subunit of PP2A binds both active and inactive PP2A. After washing away unbound material, a synthetic phosphopeptide substrate is added that is dephosphorylated by active PP2A to generate free phosphate and phosphorylated peptide. The free phosphate is detected by a sensitive dye-binding assay using malachite green and molybdic acid. By calculating the rate of phosphate release, the activity of PP2A is determined. As a positive control, we used total lysate, and as negative controls we used lysate stimulated with the PP2A/B specific phosphatase inhibitor, Okadaic acid (100 nM) (Cell signaling technology, #5934). As a control for immunoprecipitation, we used over-expression of PPP2CA in absence of Flag-GR.

### Reagents

Recombinant mouse and human TNF was produced in *E. coli* and purified to homogeneity in our laboratories. Mouse TNF and human TNF had a specific activity of 1.2 × 10^8^ IU/mg or 3.7 × 10^7^ IU/ml, respectively, and no detectable endotoxin contamination. Dexamethasone, Progesterone and GW7647 were purchased from Sigma.

### Statistical analysis

Data are expressed as the mean ± S.E. Student’s t-tests (for comparisons between two groups) or one-way ANOVA analysis (for comparisons of ≥ 3 groups) followed by Tukey’s post hoc test was used for the statistical analyses, with 95% confidence intervals and with unpaired two-tailed analysis of variance. Error bars in the figures represent the mean ± S.E. *, **, *** represent *p *< 0.05, *p *< 0.01 and *p *< 0.001, respectively.

## Electronic supplementary material

Supplementary Information
