# Supplementary material for: Glucocorticoid Receptor-mediated transactivation is hampered by Striatin-3, a novel interaction partner of the receptor
Source: Sci Rep. 2017 Aug 21;7:8941. doi: 10.1038/s41598-017-09246-6 (PMC5567040; doi:10.1038/s41598-017-09246-6)
Supplement: Supplementary file 1 — Supplementary Information [file 41598_2017_9246_MOESM1_ESM.pdf]

Supplementary information

Title

Glucocorticoid Receptor-mediated transactivation is hampered by Striatin-3, a novel interaction partner of the receptor

Ioanna Petta[a,b,c,d], Nadia Bougarne[a,b,e,¥], Jolien Vandewalle[c,d,¥], Lien Dejager[c,d], Sofie Vandevyver[c,d], Marlies Ballegeer[c,d], Sofie Desmet[a,b,e], Jonathan Thommis[a,b,e], Lode De Cauwer,[a,b,e], Sam Lievens[a,b], Claude Libert[c,d,#], Jan Tavernier[a,b,f,#], Karolien De Bosscher[a,b,e,f,#]*

[a] Receptor Research Laboratories, Cytokine Receptor Lab, VIB, Medical Biotechnology Center, Ghent, Belgium
[b] Department of Biochemistry, Ghent University, Ghent, Belgium
[c] Inflammation Research Center, VIB, Ghent, Belgium
[d] Department of Biomedical Molecular Biology, Ghent University, Ghent, Belgium
[e] Receptor Research Laboratories, Nuclear Receptor Lab, VIB, Medical Biotechnology Center, Ghent, Belgium
[f] Cancer Research Institute Ghent (CRIG), Ghent, Belgium

[#] C. Libert, J. Tavernier and K. De Bosscher contributed equally.
[¥] N. Bougarne and J. Vandewalle contributed equally.
* Correspondence to Karolien De Bosscher. E-mail: karolien.debosscher@vib-ugent.be.

Supplementary S1

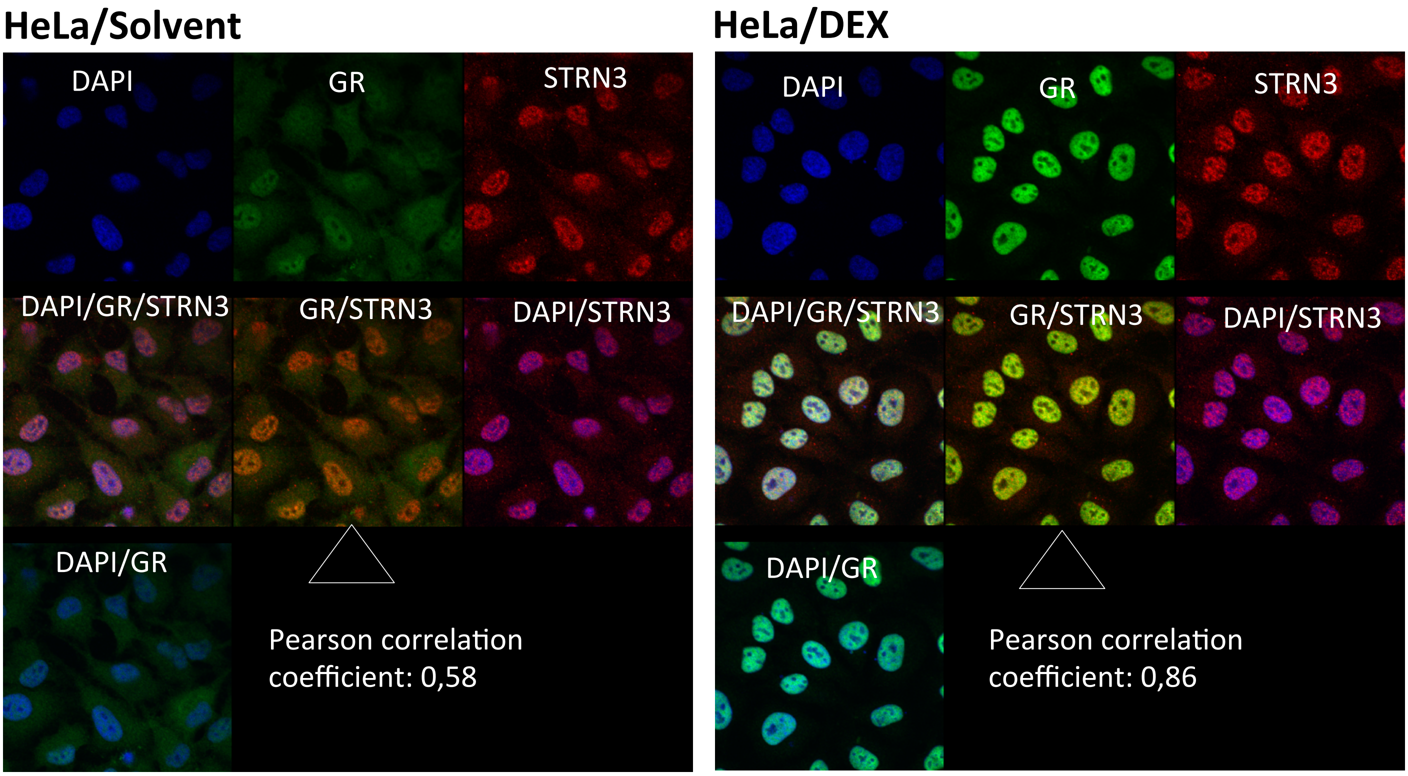

Supplementary S1. DEX treatment of HeLa cells supports co-detection of endogenous GR and STRN3 in the nucleus. HeLa cells were seeded on coverslips and incubated in phenol-red free and serum-free medium for 4h. Cells were treated with either solvent or 1 μM of DEX, for 2h following by indirect immunofluorescence analysis to detect GR (green signal) and/or STRN3 (red signal). Cell nuclei are visualized using DAPI (blue signal). Assessment of colocalization of the protein signal for GR and STRN (middle frame) was performed by correlation statistics using the Olympus Fluoview version 4.2 software. The image is a representative result of at least 3 recorded images, with each field containing minimally 10 cells.

Supplementary S2

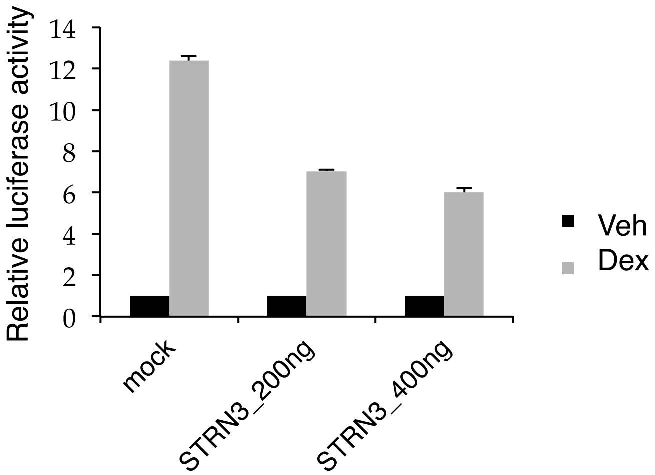

Supplementary S2. Over-expression of STRN3 decreases GR's activity in HeLa cells. HeLa cells were transfected in 24-well plate with STRN3 plasmid, in concentration of 200ng and 400ng or with mock plasmid, as indicated. The cells were treated with vehicle (black bars) or 1μM Dex (grey bars) for 6h and then lysed for measurement of luciferase activity.

Supplementary S3

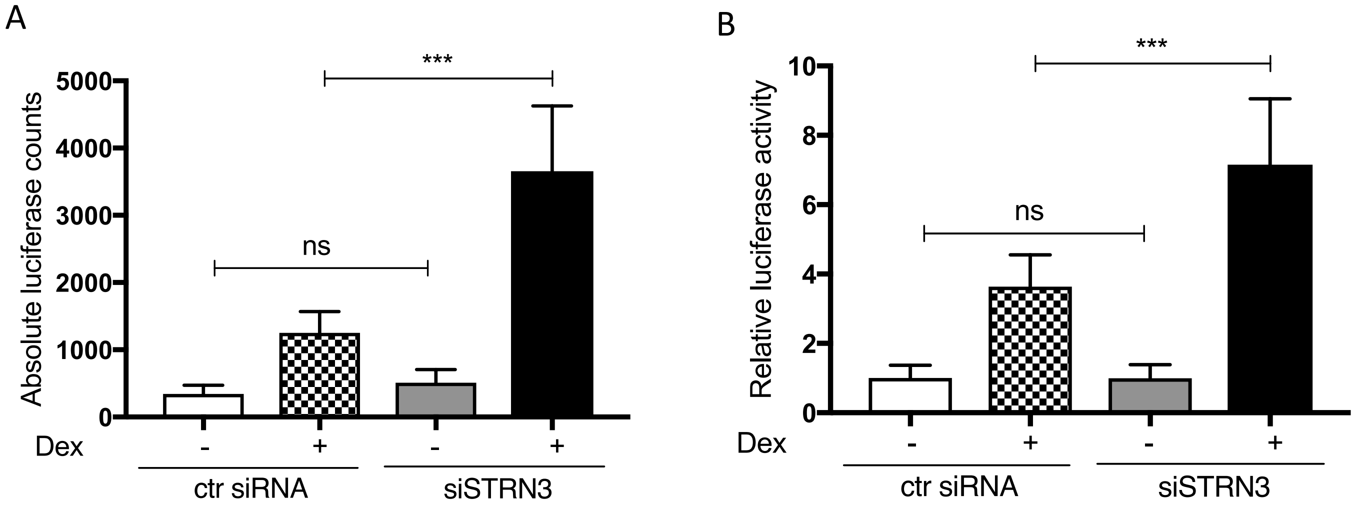

Supplementary S3. Silencing of STRN3 with siGenome siRNA. A549 cells stably transfected with GRE-Luc reporter were transfected with 50nM siRNA siGenome (Dharmacon) for human STRN3. Cells were treated with 1μM Dex or vehicle for 24h and processed for luciferase measurement. Silencing of STRN3 enhances the transcriptional activity of GR, as observed in Figure 3B and C. Here an alternative siRNA is used in order to confirm specificity and that our data are reproducible. In line with the data presented in Figure 3, silencing of STRN3 does not affect the activity of the receptor in the absence of ligand. (A) Absolute counts of the luciferase activity and (B) fold change luciferase activity normalized to the untreated (-) condition.

Supplementary S4

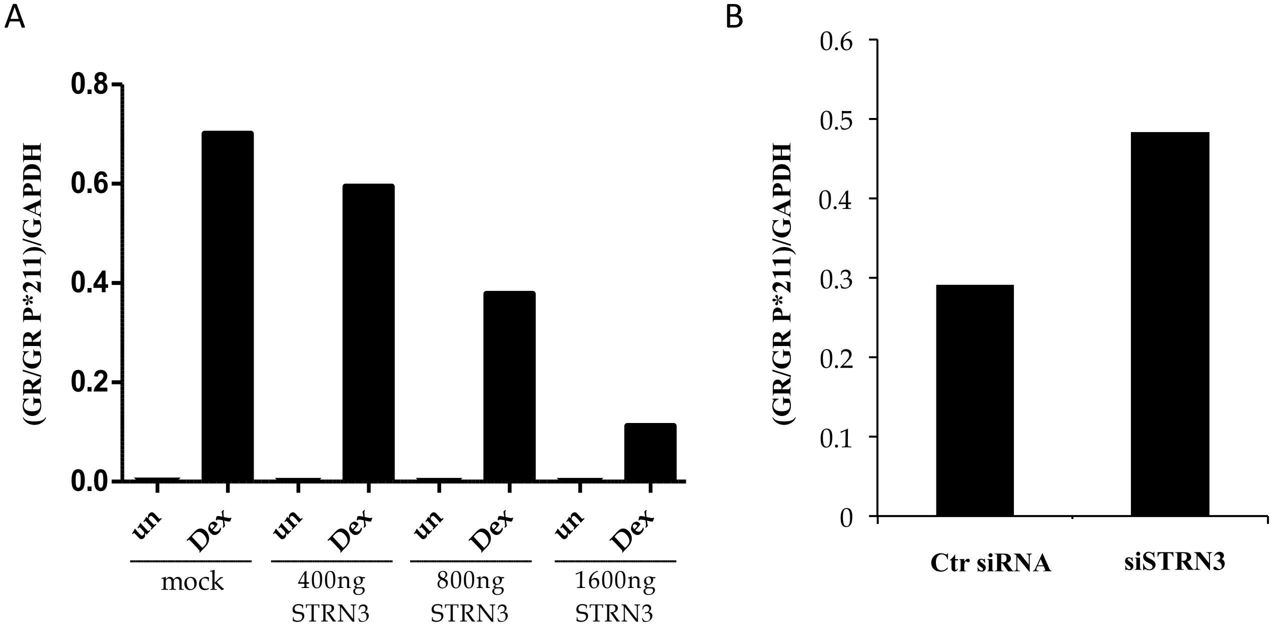

Supplementary S4. Quantification of bands of western blots of Figure 5B and C. (A) The bands of phosphorylated GR upon over-expression of mock or different concentrations of flag-STRN3 in A549 cells are quantified and normalized to total GR and to GAPDH. **(B)** The bands of phosphorylated GR upon treatment of A549 cells with ctr siRNA or siSTRN3 are quantified and normalized to total GR and to GAPDH. Statistics were not performed in these graphs because they represent results of one representative experiment out of three performed.

Supplementary S5

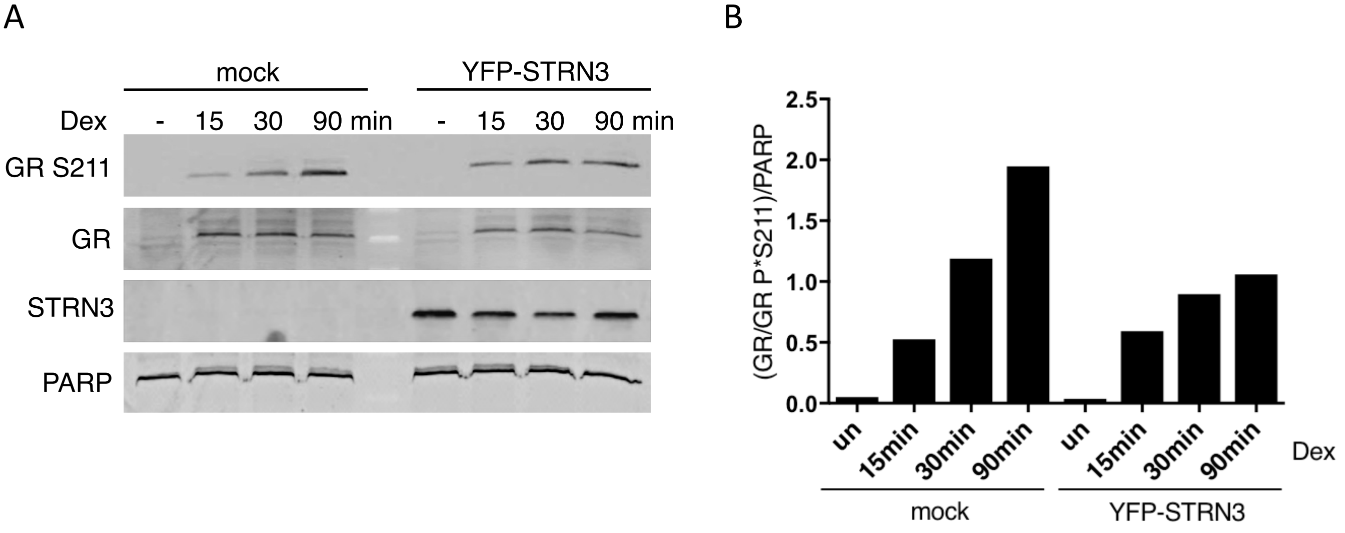

Supplementary S5. STRN3 over-expression affects GR phosphorylation on a time-dependent manner. **(A)** A549 cells transfected with 1600ng of YFP-STRN3 were stimulated with vehicle (-) or 1μM Dex (+) for different time points as indicated. Nuclear lysates were used for immunoblotting against GR phosphorylated at S211, total GR, STRN3 and PARP (nuclear marker). The immunoblots depict a representative experiment out of three independent experiments. STRN3 detection was done in a separate immunoblot to avoid multiple stripping of the membrane. **(B)** The bands of the phosphorylated GR of blot in (A) quantified and normalized to total GR and to PARP. We observe that already after 30min of Dex stimulation, over-expressed STRN3 negatively affects GR phosphorylation at S211. This effect is also observed after 90 min of Dex, indicating a time-dependent pattern of STRN3 on GR phosphorylation. Statistics were not performed in these graphs because they represent results of one representative experiment out of three performed.

Supplementary S6

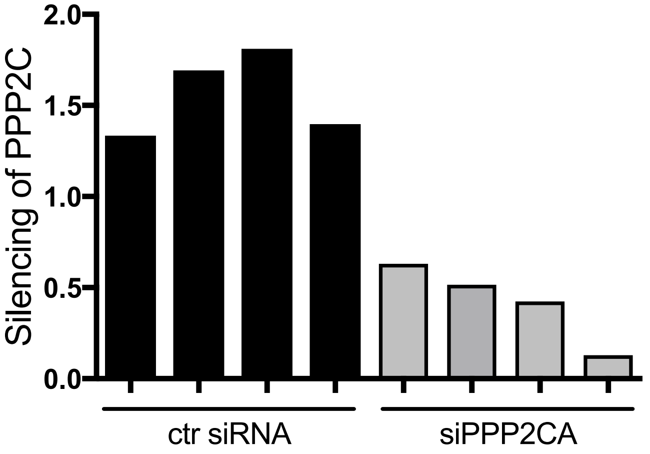

Supplementary S6. Quantification of PPP2CA silencing of figure 7B. Black bars represent the samples in lanes 1-4 that were treated with ctr siRNA and grey bars represent the samples of lanes 5-8 that were treated with siPPP2CA. PPP2CA bands were normalized to PARP. We observed an efficient level of PPP2CA silencing over 60%.

Supplementary S7

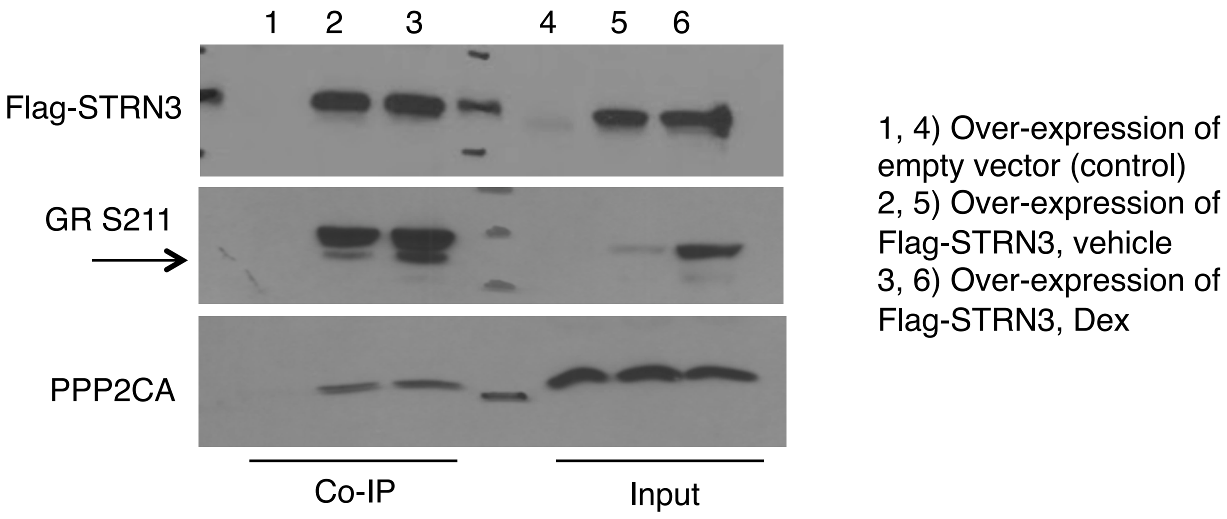

Supplementary S7. Semi-endogenous co-immunoprecipitation with Flag-STRN3 over-expression in total lysates of A549 cells. A549 cells were transfected with Flag-STRN3 or empty vector, as indicated (total amount of DNA transfected is 10μg) in 10cm plates. The cells were treated with vehicle or 1μM Dex for 2h then lysed and processed for co-immunoprecipitation with M2-Flag beads (as described in the methods section). The blots were incubated with the following antibodies: Flag, phospho GR and PPP2CA. Endogenous phosphorylated GR is precipitated together over-expressed Flag-STRN3 and endogenous PPP2CA (lanes 2 and 3)

Supplementary S8

```
GR          151 PTEKEFPKTHSDVSSEQQHLKGQTGTNGGNVKLYTTDQSTFDILQDLEFS    200

ESR1          1 --------------------------------------------------      0

GR          201 SGSPGKETNESPWRSDLLIDENCLLSPLAGEDDSFLLEGNSNEDCKPLIL    250

ESR1          1 --------------------------------------------------      0

GR          251 PDTKPKIKDNGDLVLSSPSNVTL-----------------------PQVK    277
                                  ..:||                       ||:|
ESR1          1 ------------------MTMTLHTKASGMALLHQIQGNELEPLNRPQLK     32

GR          278 TEKEDFIELCTPGVIKQEKLGTVYCQASFPGANIIGNKMSAISVHGVSTS    327
                ...|             ..||.||..:|.|..              .:..
ESR1         33 IPLE-------------RPLGEVYLDSSKPAV--------------YNYP     55

GR          328 GGQMYHYDMNTASLSQQQDQKPIFNVIPPIPVGSENWNRCQGSGDDNLTS    377
                .|..|.::...|:.:|...|..:     |...|||    ....|.:.|..
ESR1         56 EGAAYEFNAAAAANAQVYGQTGL-----PYGPGSE----AAAFGSNGLGG     96

GR          378 LGTLN----FPGRTVFSNGYSSPSMRPD-------VSSPPSSSSTATTGP    416
                ...||    .|...:......||.::|.       :.:.||..:....||
ESR1         97 FPPLNSVSPSPLMLLHPPPQLSPFLQPHGQQVPYYLENEPSGYTVREAGP    146

GR          417 P----------------------------------PKLCLVCSDEASGCH    432
                |                                  .:.|.||:|.|||.|
ESR1        147 PAFYRPNSDNRRQGGRERLASTNDKGSMAMESAKETRYCAVCNDYASGYH    196

GR          433 YGVLTCGSCKVFFKRAVEGQHNYLCAGRNDCIIDKIRRKNCPACRYRKCL    482
                |||.:|..||.||||:::|.::|:|...|.|.|||.|||:|.|||.|||.
ESR1        197 YGVWSCEGCKAFFKRSIQGHNDYMCPATNQCTIDKNRRKSCQACRLRKCY    246

GR          483 QAGMN----LEARKTKKKIKGIQQATTGVSQETSENPG------------    516
```

Supplementary S8. Protein alignment of human Glucocorticoid receptor α (GR) and human Estrogen Receptor α (ESR1). GR S211 (red box) does not correspond to ESR1 S118 (blue box) for which a similar action of STRN3 and PP2CA has been proposed. Vertical lines indicate identical amino acids. Dotted or double-dotted connections indicate degree of relevance between different amino acids in the two protein sequences.

Supplementary S9

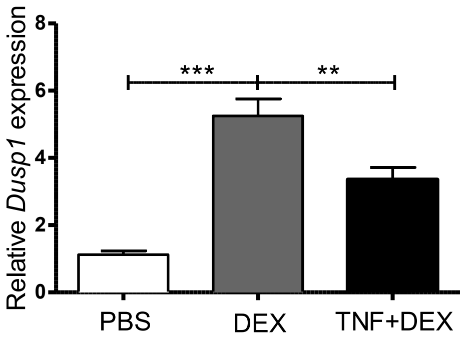

Supplementary S9. TNF inhibits GR transactivation capacity. C57BL/6 mice were injected with PBS (white bar, n=4) or 200µg Dex (grey bar, n=4) or with 15µg of TNF 1h prior to 5mg/kg of Dex injection (black bar, n=4). 6h after Dex injections the mice were killed with cervical dislocation and a piece of their liver was used for RNA isolation. These samples were used for qPCR to measure the endogenous levels of *DUSP1*. **, $p < 0.01$, ***, $p < 0.001$

Supplementary S10

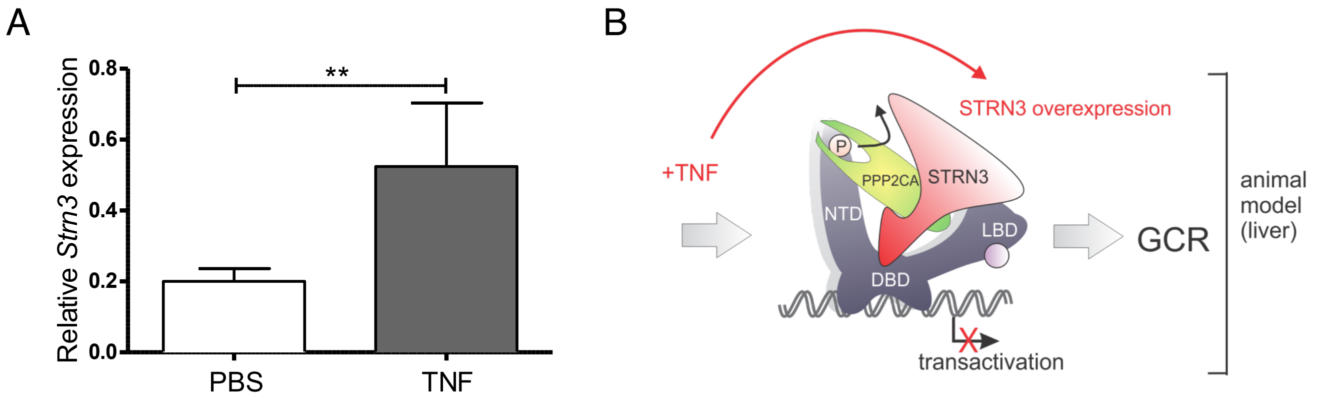

Supplementary S10. A hypothesis model for the involvement of STRN3 in the TNF-induced GCR. (A) WT C57BL/6 mice were injected with PBS (white bar, n=4) or with 15µg of TNF (grey bar, n=4). 6h after injections, the mice were killed and a piece of their liver was used for RNA isolation and qPCR to measure endogenous Strn3 levels. **(B)** TNF-induced increase of STRN3 levels may contribute to glucocorticoid resistance by an imbalanced and ongoing PPP2CA recruitment to the GR complex. GR with its protein domains (NTD, DBD, LBD) is depicted in grey. P stands for phosphorylated S211. The purple circle represents the ligand, Dex. GCR; Glucocorticoid resistance. TNF; Tumor necrosis factor. **, $p < 0.01$.

Mice injections and sampling: Normal female C57BL/6 mice, purchased from Janvier Labs, were aged 8–10 weeks. Mice were kept in an SPF, air-conditioned and light controlled animal house and received food and water *ad libitum*. 15µg TNF in 100µl PBS were injected intraperitoneally 30min prior to 5mg/kg Dexamethasone. TNF or Dex injections were performed in a total volume of 200µl. Mice were killed by cervical dislocation. A piece of liver was stored in RNA later (Qiagen) for RNA preparation. Experimental procedures were carried out in accordance with the approved guidelines by the institutional ethics committee of animal welfare of the faculty of sciences, Ghent University, Belgium (ethical file number 2014-007)

Supplementary S11

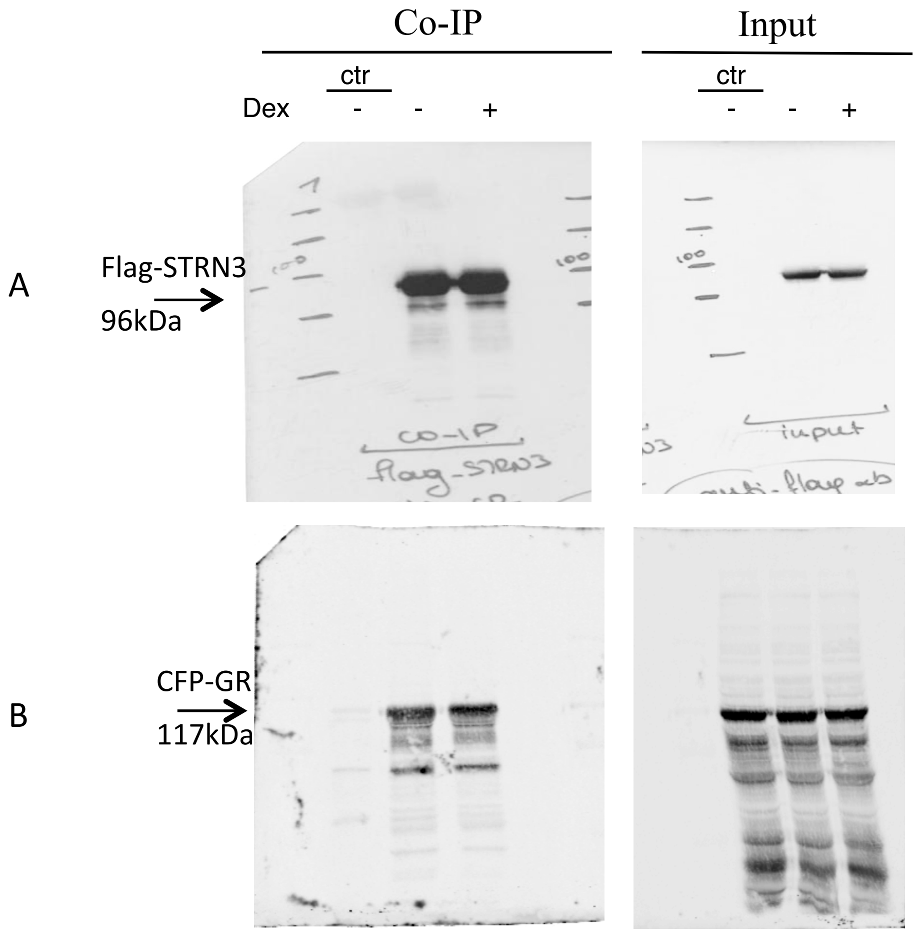

Supplementary S11. Full-length Western blots of figure 1D. (A) Immunoprecipitation of Flag-STRN3 with M2 Flag beads (left panel) and detection of Flag-STRN3 in input samples (right panel). Detection was done with anti-Flag antibody and Odyssey. (B) Co-immunoprecipitation of CFP-GR. Detection of CFP-GR was done with anti-GR antibody and Odyssey. Information about the primary and secondary antibodies are provided in the methods section.

Supplementary S12

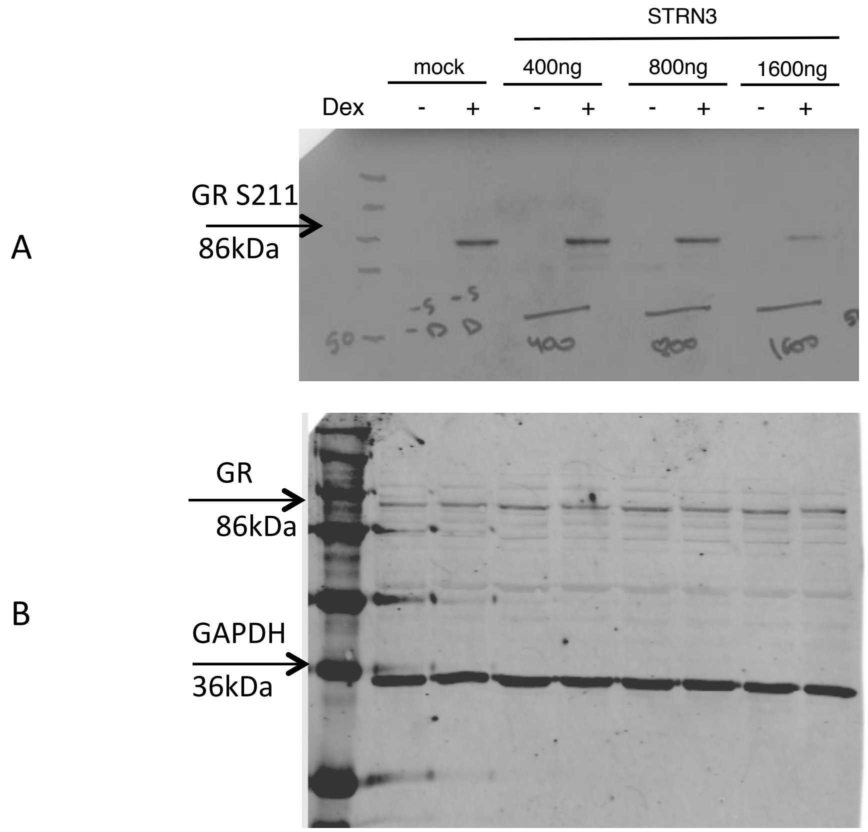

Supplementary S12. Full-length Western blots of figure 5B. Total A549 lysates were analyzed for the expression of: (A) phosphorylated GR S211 with ECL, (B) total GR and GAPDH with Odyssey. Information about the primary and secondary antibodies are provided in the methods section.

Supplementary S13

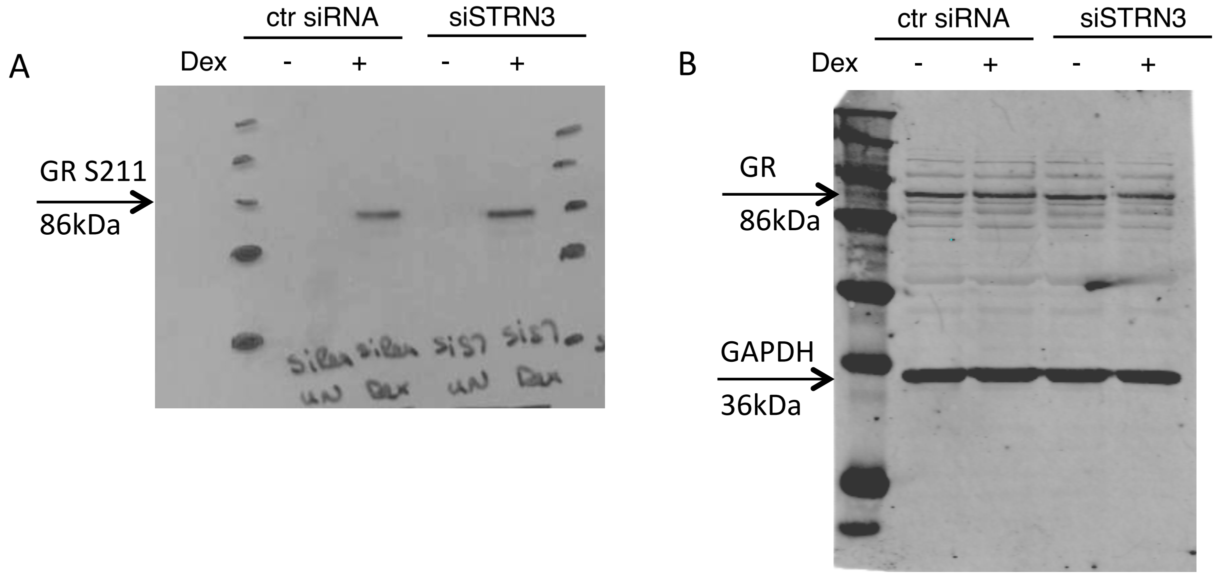

Supplementary S13. Full-length Western blots of figure 5C. Total A549 lysates were analyzed for the expression of: (A) phosphorylated GR S211 with ECL, (B) total GR and GAPDH with Odyssey. Information about the primary and secondary antibodies are provided in the methods section.

Supplementary S14

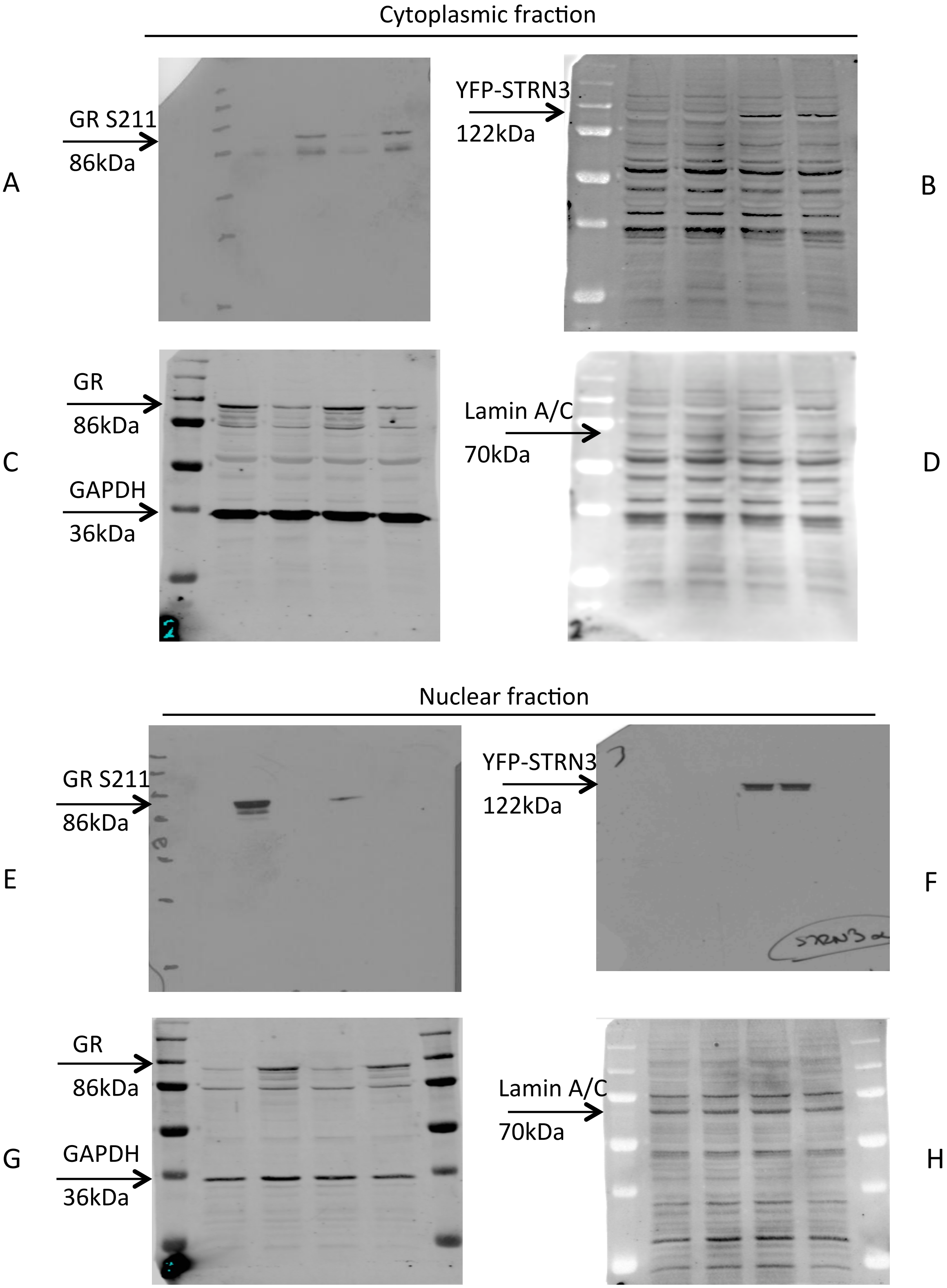

Supplementary S14. Full-length Western blots of figure 5E. A549 lysates separated in cytoplasmic and nuclear fractions. The cytoplasmic fraction was analyzed for the expression of: (A) phosphorylated GR S211 with ECL, (B) YFP-STRN3 with Odyssey (C) total GR and GAPDH with Odyssey, (D) Lamin A/C. The cytoplasmic fraction was analyzed for the expression of: (E) phosphorylated GR S211 with ECL, (F) YFP-STRN3 with Odyssey, (G) total GR and GAPDH with Odyssey, (H) Lamin A/C with Odyssey. Information about the primary and secondary antibodies are provided in the methods section.

Supplementary S15

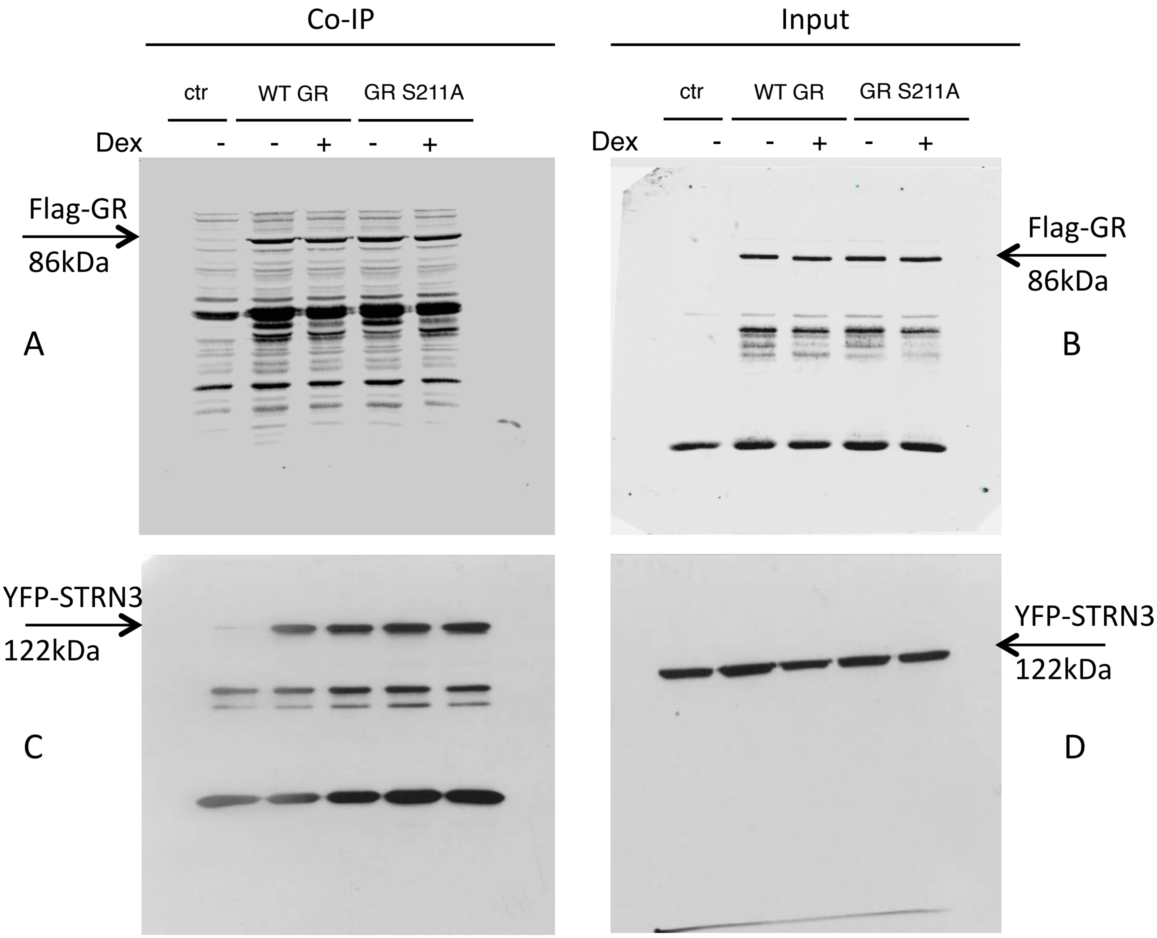

Supplementary S15. Full-length Western blots of figure 6B. Co-immunoprecipitation (A, C) and input blots (B, D). (A) Immunoprecipitation of WT Flag-GR or Flag-GR mutant S211A with M2 Flag beads. (B) Detection of WT Flag-GR and Flag-GR mutant S211A over-expression in input samples. In A and B detection was done with anti-Flag antibody and Odyssey. (C) Co-immunoprecipitation of YFP-STRN3 with Flag-GR WT and GR mutant S211A. (D) Detection of YFP-STRN3 over-expression in input samples. In C and D detection was done with anti-STRN3 antibody and ECL. Information about the primary and secondary antibodies are provided in the methods section.

Supplementary S16

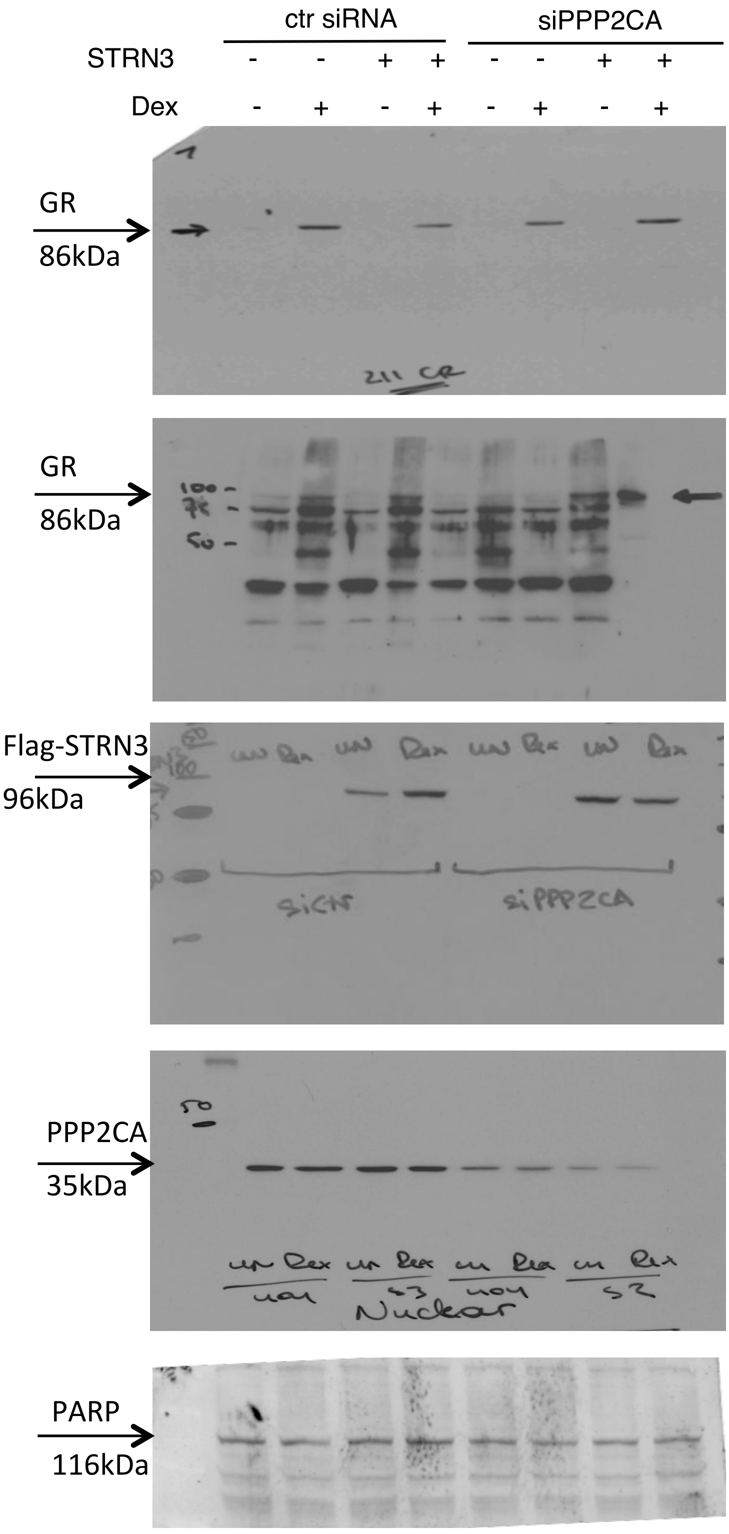

Supplementary S16. Full-length Western blots of figure 7B. A549 nuclear lysates were analyzed for the expression of: (A) phosphorylated GR S211 (95kDa) with ECL (B) Total GR (95kDa) with ECL (in a separate western blot) (C) Flag-STRN3 with ECL with anti-Flag antibody (D) Endogenous PPP2CA with ECL (siPPP2CA used in lanes 5-8) (E) PARP with Odyssey. Information about the primary and secondary antibodies are provided in the methods section.

Supplementary S17

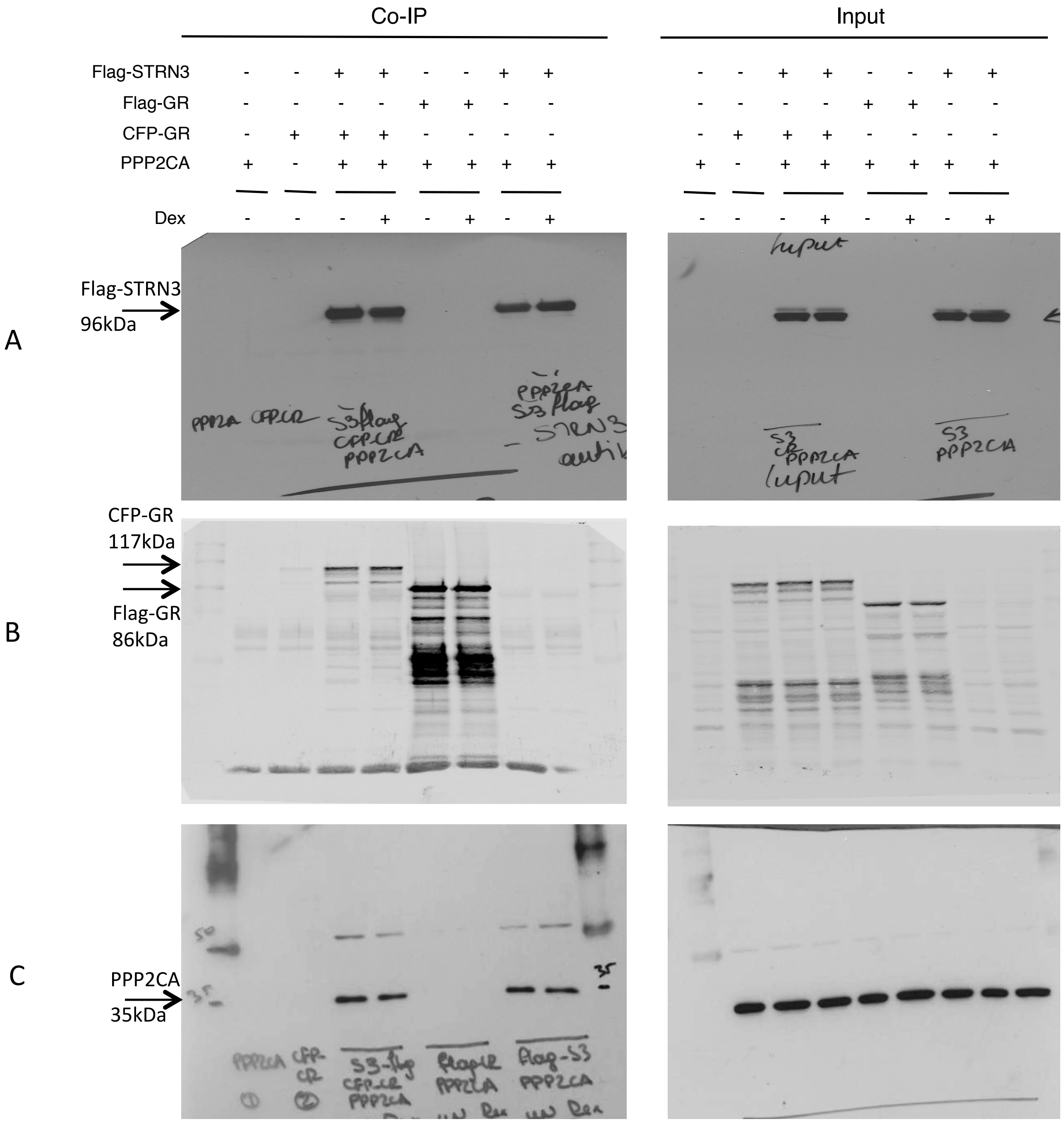

Supplementary S17. Full-length Western blots of figure 7C. Immunoprecipitation with M2-Flag beads against Flag-STRN3 and Flag-GR as indicated. The co-immunoprecipitation (left panels) and input (right panels) blots were analyzed for the detection of: (A) Flag-STRN3 with anti-STRN3 antibody with ECL, (B) CFP-GR and Flag-GR with anti-GR antibody with Odyssey, (C) PPP2CA with anti-PPP2CA antibody with ECL. Information about the primary and secondary antibodies are provided in the methods section.

Supplementary S18

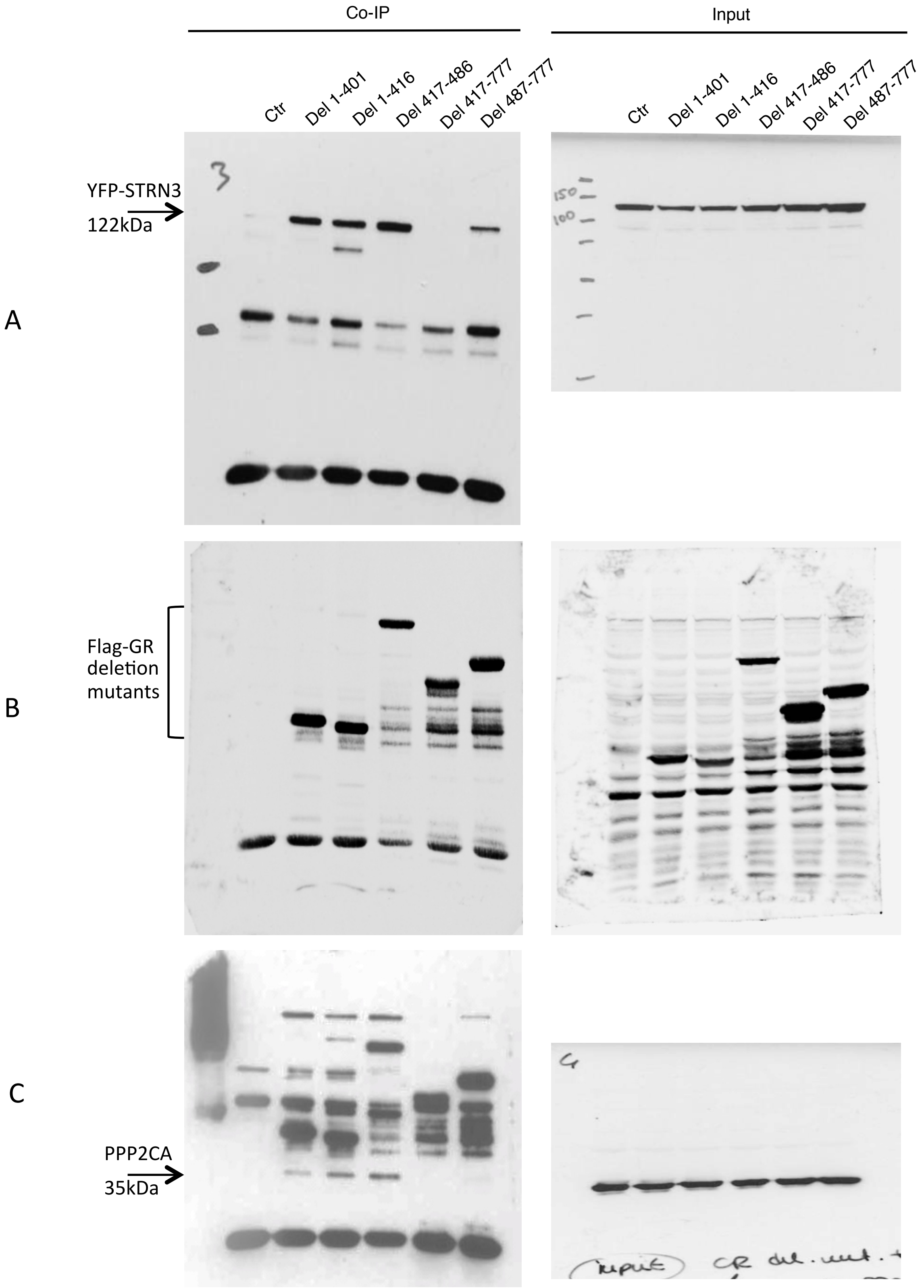

Supplementary S18. Full-length Western blots of figure 8B. Immunoprecipitation with M2-Flag beads against Flag-GR deletion mutants. The co-immunoprecipitation (left panels) and input (right panels) blots were analyzed for the detection of: (A) YFP-STRN3 with anti-STRN3 antibody with ECL, (B) Flag-GR deletion mutants with anti-Flag antibody with Odyssey, (C) endogenous PPP2CA with anti-PPP2CA antibody with Odyssey (left panel) and ECL (right panel). Information about the primary and secondary antibodies are provided in the methods section.

Molecular weights of GR deletion mutants. Del1-401: 42kDa, Del1-416: 40kDa, Del417-486: 78kDa, Del417-777: 46kDa, Del487-777: 54kDa.
